# Apolipoprotein L1 (APOL1) renal risk variant-mediated podocyte cytotoxicity depends on African haplotype and surface expression

**DOI:** 10.1038/s41598-024-53298-4

**Published:** 2024-02-14

**Authors:** Nidhi Gupta, Bridget Waas, Daniel Austin, Ann M. De Mazière, Pekka Kujala, Amy D. Stockwell, Tianbo Li, Brian L. Yaspan, Judith Klumperman, Suzie J. Scales

**Affiliations:** 1 Department of Discovery Immunology, Genentech, 1 DNA Way, South San Francisco, CA 94080 USA; 2 Department of Molecular Biology, Genentech, 1 DNA Way, South San Francisco, CA 94080 USA; 3 Department of Biochemical and Cellular Pharmacology, Genentech, 1 DNA Way, South San Francisco, CA 94080 USA; 4 Section of Cell Biology, Center for Molecular Medicine, University Medical Center Utrecht, Utrecht University, Utrecht, The Netherlands; 5 Department of Human Genetics, Genentech, 1 DNA Way, South San Francisco, CA 94080 USA; 6 Present Address: Department of Cell and Developmental Biology, University of Michigan, Ann Arbor, MI 48109 USA

**Keywords:** Chronic kidney disease, Secretion, Protein transport

## Abstract

Homozygous Apolipoprotein L1 (APOL1) variants G1 and G2 cause APOL1-mediated kidney disease, purportedly acting as surface cation channels in podocytes. APOL1-G0 exhibits various single nucleotide polymorphisms, most commonly haplotype E150K, M228I and R255K (“KIK”; the Reference Sequence is “EMR”), whereas variants G1 and G2 are mostly found in a single “African” haplotype background (“EIK”). Several labs reported cytotoxicity with risk variants G1 and G2 in KIK or EIK background haplotypes, but used HEK-293 cells and did not verify equal surface expression. To see if haplotype matters in a more relevant cell type, we induced APOL1-G0, G1 and G2 EIK, KIK and EMR at comparable surface levels in immortalized podocytes. G1 and G2 risk variants (but not G0) caused dose-dependent podocyte death within 48h only in their native African EIK haplotype and correlated with K^+^ conductance (thallium FLIPR). We ruled out differences in localization and trafficking, except for possibly greater surface clustering of cytotoxic haplotypes. APOL1 surface expression was required, since Brefeldin A rescued cytotoxicity; and cytoplasmic isoforms vB3 and vC were not cytotoxic. Thus, APOL1-EIK risk variants kill podocytes in a dose and haplotype-dependent manner (as in HEK-293 cells), whereas unlike in HEK-293 cells the KIK risk variants did not.

## Introduction

Apolipoprotein L1 (APOL1) circulates in the blood and protects humans against infection by African trypanosomes^1^. APOL1 variants G1 (S342G, I384M) and G2 (∆N388:Y389) evolved to additionally protect from *Trypanosoma Brucei Gambiense* and *Rhodesiense* and are thus highly prevalent in sub-Saharan Africa and the African diaspora^2,3^. People carrying two copies of the variants (including ~ 6 million African Americans) harbor around 70% excess risk of non-diabetic APOL1-mediated kidney disease (AMKD)^4^.

Enormous efforts have been made over the last decade to understand the mechanism of action of the APOL1 variants in causing kidney damage. The prevailing hypothesis that APOL1 risk variant-driven cation influx likely drives podocyte cytotoxicity is strongly supported by the impressive decrease in proteinuria of African Americans with focal segmental glomerulosclerosis (FSGS) in a phase II trial of Vertex’s small molecule inhibitor of APOL1 channel function (VX-147, Inaxaplin)^5^. APOL1-G1 and G2 should conduct more cations than G0 to explain their role in AMKD. However, while in vitro studies with recombinant APOL1 in liposomes suggest G1 and G2 conduct more K^+^ ions than G0, these results were partially confounded by greater liposomal insertion of G1 and G2^6,7^.

**Figure 1:**
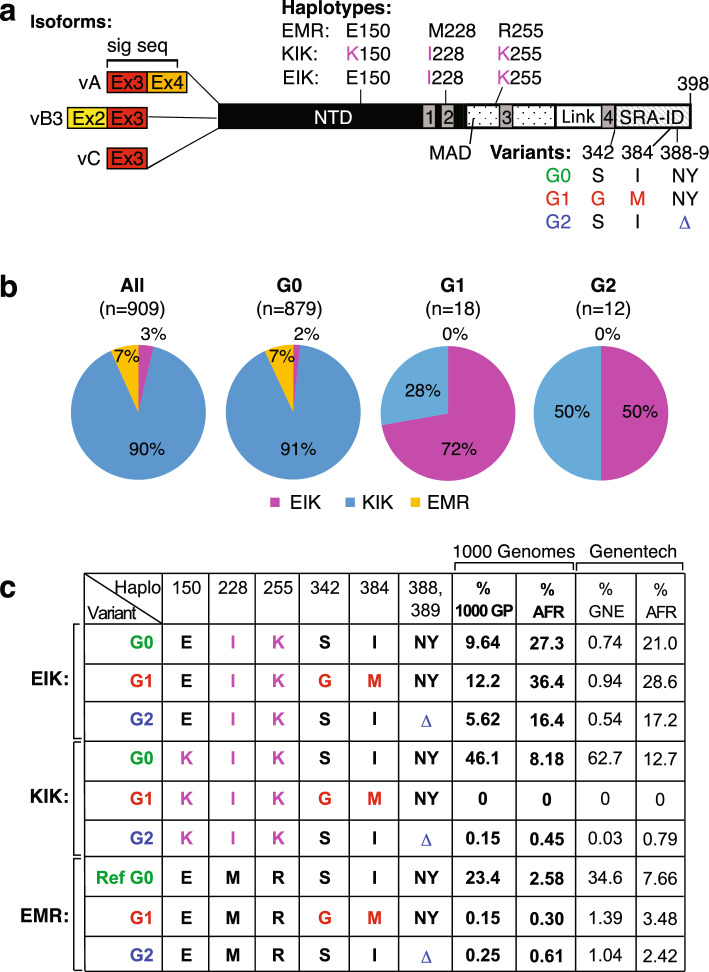
APOL1 haplotypes and their frequencies. (**a**) SNP positions in the APOL1 protein. The E150K SNP is in the N-terminal domain (NTD^18^, previously called the Pore Forming Domain); M228I is in the 2nd putative transmembrane domain (grey rectangles numbered 1–4; the 3rd one is not predicted by all models)^19^; and R255K is in the membrane addressing domain (MAD; dotted) just before the tentative third transmembrane domain. The renal risk variants, G1 and G2, are in the SRA-interacting domain (SRA-ID; hatched). Linker (Link) denotes the region (between transmembrane domains 3 and 4) that is hypothesized by Schaub et al*.* to be intracellular in the active conformation of the APOL1 cation channel^20^. The N-terminal splice isoforms with amino acids encoded by exon 2 (Ex2) in yellow, exon 3 (Ex3) in red and exon 4 (Ex4) in orange. The signal sequence encoded by exons 3 and 4 is disrupted in isoforms vB3 and vC, which lack exon 4 (see Supplementary Table S1 for complete sequences and SNP rs numbers). (**b**) Pie charts showing percentages of 909 sequenced cell lines in the Genentech cell line repository with APOL1 EIK (magenta), KIK (blue) or EMR (orange) haplotypes of G0, G1 (14 African, 1 Caucasian, 1 Admixed African and 2 unknown) or G2 (all 12 African) variants. Note some cell lines also possessed other SNPs (e.g. G96R, N264K) not categorized separately for simplicity. (**c**) Frequencies of the haplotypes investigated herein in human samples. Frequencies in the 1000 Genomes (% 1000 GP) phase 3 population (*n* = 2012 unrelated samples only), overall (% total, bold) and prevalence within the African superpopulation (% AFR, bold). Non-bold numbers indicate prevalence within a much larger (*n* = 32,716 unrelated patients), more European-biased Genentech dataset (% GNE) and percentages of their AFR superpopulation. See Supplementary Table S2 for calculations and prevalence in other superpopulations. The haplotypes refer to amino acids at positions 150, 228 and 255 with changes from the RefSeq (EMR; NM_003661) in magenta; G1 variant is in red and G2 deletion (Δ) is in blue.

A commonly used cell model to examine the cytotoxic mechanisms of APOL1 variants are HEK-293 cells. In some cases, APOL1-G1 and G2 expression elicited greater cytotoxicity than G0^8–12^, but not in others^13,14^. Lannon et al.^15^ demonstrated that the APOL1 background haplotype affects cytotoxicity, with G1 and G2 risk variants being more toxic than G0 in the EIK or KIK backgrounds, and less so in the EMR background (the letters represent amino acids at positions 150, 228 and 255, respectively, of the 398aa isoform vA; Fig. 1a). Notably, perhaps because HEK-293 cells do not normally express APOL1, G1/G2-KIK and G0-EIK haplotypes caused cytotoxicity^8,10,15^, whereas AMKD is associated with G1 and G2 that are almost exclusively in the EIK haplotype. Furthermore, several labs use APOL1-G0 *K*IK as a control for G1 and G2-*E*IK, unfairly enhancing the apparent risk variant activity^9,10,12^. Measurements of cation flux have also been performed in APOL1-HEK-293 cells, but either used the EMR haplotype for all variants^13^, a mis-matched haplotype for the G0 control^9,10,16^ or undefined haplotypes^17^. It also remains to be demonstrated whether cation conductance actually correlates with cytotoxicity for all the haplotypes in the relevant cell type, podocytes.

Another issue is that cytotoxicity is clearly dependent on APOL1 expression levels^10,13,21^ and the majority of reports (including Lannon et al.)^8,10–13,15,17^ show total APOL1 expression, rather than APOL1 levels at the plasma membrane, where cation channel activity occurs^9,14^. This is potentially important because APOL1 variants may fold differently compared to G0^22,23^ and might thus be differentially retained in the endoplasmic reticulum versus being transported to the cell surface^11^.

To resolve these issues, we expressed APOL1-G0, G1 and G2 with EIK, KIK, and EMR haplotypes in physiologically relevant immortalized human podocytes^24^, and attempted to select clones with equal surface expression to compare cytotoxicity. This also enabled us to test an earlier proposal that E150K might be protective in African Americans^25^, a genetic hypothesis confounded by the lower prevalence of G1/G2 on the KIK vs EIK haplotype^26^. We found APOL1-G1 and G2 were only toxic in their native “African” EIK background, with G0-EIK being far slower to kill and the three KIKs barely toxic at all. We correlated transport kinetics with swelling and cytotoxicity, and could prevent killing with the secretion inhibitor Brefeldin A or cytoplasmic expression (of vB3 and vC isoforms), supporting the need for APOL1 to reach the plasma membrane to elicit toxicity. We also used our antibodies to look at APOL1 on the podocyte surface, finding G1 and G2-EIK appear to cluster more readily than the non-toxic haplotypes, implying greater oligomerization potential into active channels that could explain their greater cation conductance as monitored by FLIPR.

## Results

### APOL1 G1 and G2 exist on all three haplotype backgrounds

APOL1 exhibits various SNPs along its length besides G1 and G2^26–29^. Herein we refer to APOL1-G0, G1 and G2 as variants; the other (more N-terminal) SNPs are referenced as haplotypes (EIK, KIK and EMR); and the splice variants (vA, vB3 and vC) are termed isoforms (Fig. 1a, Supplementary Table S1). The first draft of the 1000 Genomes Project showed APOL1-G1 and G2 risk variants solely on the EIK haplotype^9,29^, which is restricted to the AFR superpopulation (Africans and their diaspora in the USA and Barbados). However, 28% (5/18) of G1 and 50% (6/12) of G2 cell lines in Genentech’s repository^30^ are KIK (Fig. 1b), suggesting the risk variants must exist on this haplotype too. By contrast, of 879 G0 cell lines, 91% were KIK, 2% were EIK and 7% were EMR. This prompted us to evaluate the 1000 Genomes phase 3 data (*n* = 2012)^31^ as well as a larger cohort of Genentech samples (*n* = 32,716) to confirm that APOL1-G2 (though not APOL1-G1 at least in these samples) does indeed exist in KIK haplotype Africans, albeit at low frequency (up 0.15% of the total and 0.45–0.79% of AFR in the two human cohorts; Fig. 1c and Supplementary Table S2). In contrast to the cell lines, there were actually more G1 and G2 on the EMR haplotype in humans, comprising up to 3.5% of AFR and 1.4% of the totals, respectively. Also notable is that in these larger datasets APOL1-G0 is 2–3 × more frequent on the EIK than KIK haplotype in the AFR population, thus the recent dubbing of G0-KIK as “AfCom” (African Common)^16^ based on 1000 Genomes phase 1 data^15^ is no longer accurate.

### APOL1 risk variant-mediated cell death is dose and haplotype dependent

**Figure 2:**
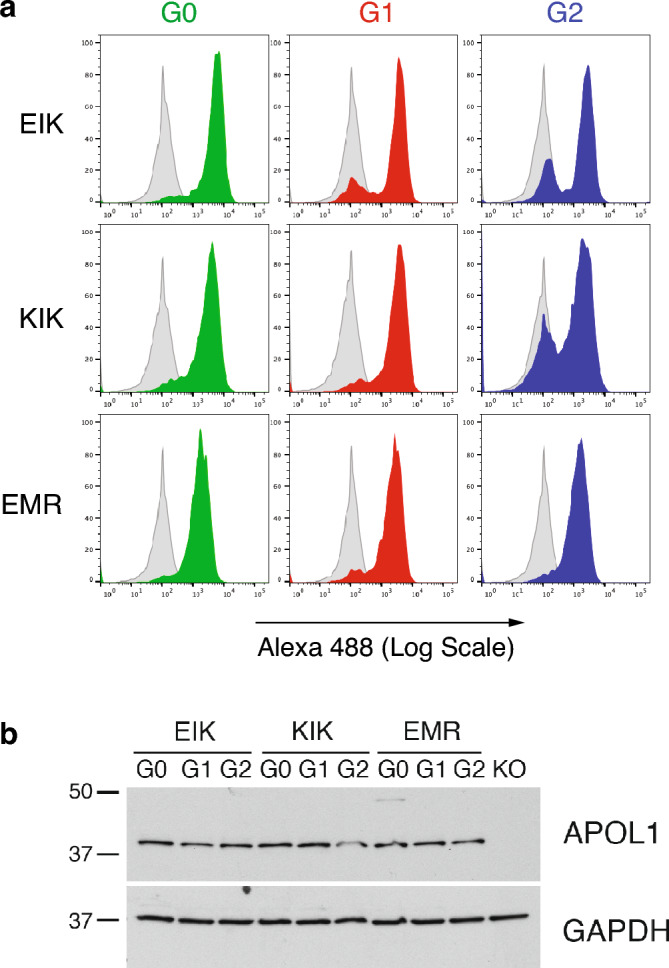
Podocytes stably expressing equal surface levels of various haplotypes of APOL1. (**a**) Flow cytometry of iAPOL1-podocytes showing similar surface expression between haplotypes. Podocyte clones of “African” EIK, world-wide common KIK or reference EMR APOL1 haplotypes were induced for 24h with 5 ng/ml dox, harvested and stained for FACS with Alexa488-conjugated anti-APOL1 monoclonal 3.7D6. This direct staining method highlights any differences in expression compared to two-step staining, which was used during the actual selection of the top clones for each haplotype (compare Supplemental Fig. 1). (**b**) Western blot of the same cell lines. APOL1 was detected in 4µg lysates on a single blot using an oligoclonal mixture of rabmabs 3.7D6 and 3.1C1, with GAPDH as a loading control (see Supplementary Fig. 22 for full length blots). This blot is representative of 4 experiments.

Since G1 and G2 do exist on other haplotypes than EIK, we wanted to examine if haplotype affects risk variant-mediated cytotoxicity in podocytes like they do in HEK-293 cells^15^. To do this, we used APOL1-knockout podocytes^32^ to avoid any potential endogenous G0 dominance^26^, and stably re-expressed the three different APOL1 haplotypes (KIK^32^, EIK and EMR) of variants G0, G1 and G2 (all the major (secretory) isoform vA)^19,33^ under a doxycycline (dox)-inducible promoter (iAPOL1 podocytes). We used flow cytometry to select single cell clones expressing similar APOL1 levels at the cell surface (Fig. 2a), because it is the likely site of action of APOL1 and more relevant in respect to ion channel activity than total levels assessed by Western blotting of cell lysates^8–10,13,15^. Western blotting of our selected clones actually showed minor differences in total protein expression (Fig. 2b).

**Figure 3:**
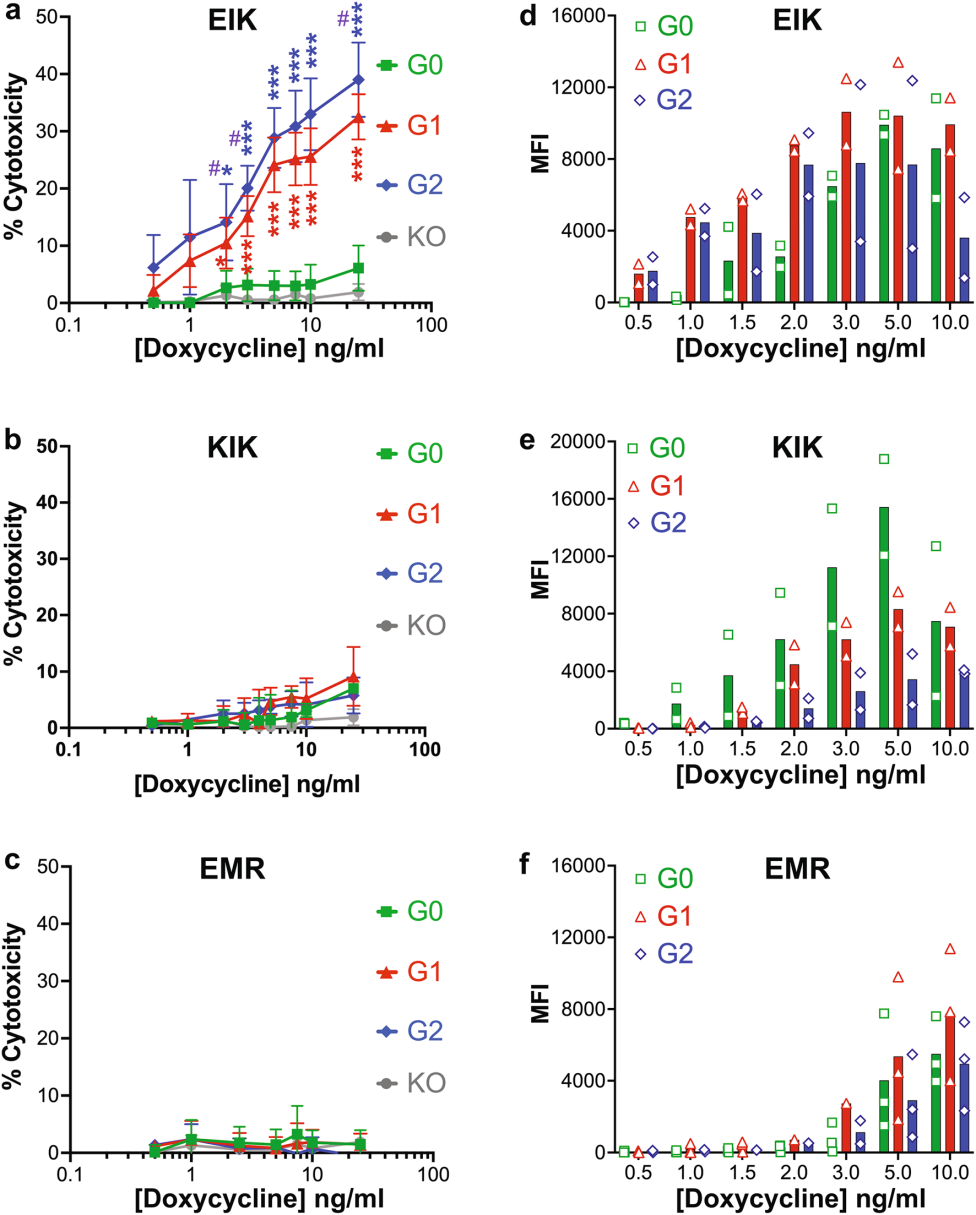
Only African EIK haplotype APOL1-G1 and G2 mediate dose-dependent podocyte cytotoxicity. (**a**-**c**) CytoTox-Glo™ assay after 48 h induction with 0-25ng/ml dox in iAPOL1 podocytes. (a) Cell death was only observed in G1 (red filled triangle) and G2 (blue filled diamond) in the EIK haplotype (mean and SD of *n* = 8 independent duplicate or quadruplicate experiments), and not with G0 (green filled square). Little or no cell death was observed in KIK (b; *n* = 6) and none in EMR (c; *n* = 5) compared to the APOL1-KO control cells (grey; *n* = 2 on all graphs). The y-axes denote the % cell death (normalized to total cell count followed by subtraction of no dox background signal). *, *p* < 0.05; ***, *p* < 0.001 vs KO; #, *p* < 0.05 for G2 vs G1. (**d**-**f**) Surface APOL1 expression by FACS after 16h induction of iAPOL1 podocytes with the indicated dox concentrations. Mean fluorescence intensities (MFI) from 2–3 different experiments are plotted as symbols, with the mean values as a bar. Note G1 and G2 EIK (d) arrive on the membrane at lower concentrations than all the others (**e**,**f**). Direct staining with Alexa488-3.7D6 was employed here to better distinguish any differences in expression levels, whereas with secondary antibody amplification, the expression appeared more homogeneous between haplotypes (see Supplementary Fig. 1).

Increased dox concentrations led to higher APOL1 expression in all clones (Supplementary Fig. 1), but APOL1-induced cell death only occurred with the G1 and G2 risk variants in the EIK haplotype (Fig. 3a). Killing was dose-dependent, starting at 1ng/ml and 0.5ng/ml of dox for G1 and G2-EIK, respectively, when expression was already substantial (Fig. 3d). APOL1-G2-EIK was slightly more cytotoxic than G1-EIK at all doses in every experiment (although inter-experimental variability largely obscured its significance), suggesting it is inherently more potent, especially considering its marginally lower surface expression. By contrast, APOL1-G0-EIK was not toxic until 2 days later and then only at higher dox concentrations (Supplementary Fig. 2a,b), despite slightly higher maximal expression than G2. By contrast, in the KIK background, only non-signficant cytotoxicity above the APOL1-KO parental control was seen with all three variants, although the peak surface expression was similar to EIK (Fig. 3b,e). KIK killing was comparable to that elicited by G0-EIK, even after 72h (Supplementary Fig. 2c). There was no cytotoxicity with any of the EMR clones at high dox even up to 120h (Fig. 3c, Supplementary Figs. 2d,e), although unfortunately none expressed as highly as EIK (Fig. 3f). Nevertheless, APOL1-G1 and G2-EIK were still toxic as low as 1ng/ml dox, when their surface APOL1 levels were below the maximal EMR levels (Fig. 3d,f), highlighting the advantage of using a titratable expression system. These data were reproduced with at least two independent clones, suggesting they are not merely single clone artifacts (Supplementary Fig. 3 and data not shown). Thus, APOL1 haplotype strongly influences G1 and G2-mediated podocyte cytotoxicity, and is dose-dependent. Our data follows similar trends to APOL1-HEK-293 cytotoxicity^10,15^, with the exceptions of the far lesser, much delayed toxicity of G0-EIK and near absence of KIK toxicity in podocytes.

### All APOL1 haplotypes and variants localize similarly.

We wanted to understand how haplotype affects the ability of G1 and G2 variants to kill podocytes. As expected from their cell surface expression, all three APOL1 variants in the EIK and EMR haplotypes were found in the secretory pathway (ER lumen, Golgi and plasma membrane) by immunofluorescence and immunoelectron microscopy (Supplementary Figs. 4–7), like the three KIK variants^19^. There were also no differences in plasma membrane topology as assessed by surface epitope exposure by FACS (Supplementary Fig. 8), including of the linker domain that was proposed to be intracellular based on the orientation of active channels in synthetic bilayers^20^. This implies there are no global differences in native APOL1 conformation in cell membranes, or that only an undetectable proportion of APOL1 channels is active (with the linker domain not exposed) at any one time.

### APOL1 reaches the plasma membrane prior to podocyte swelling.

**Figure 4:**
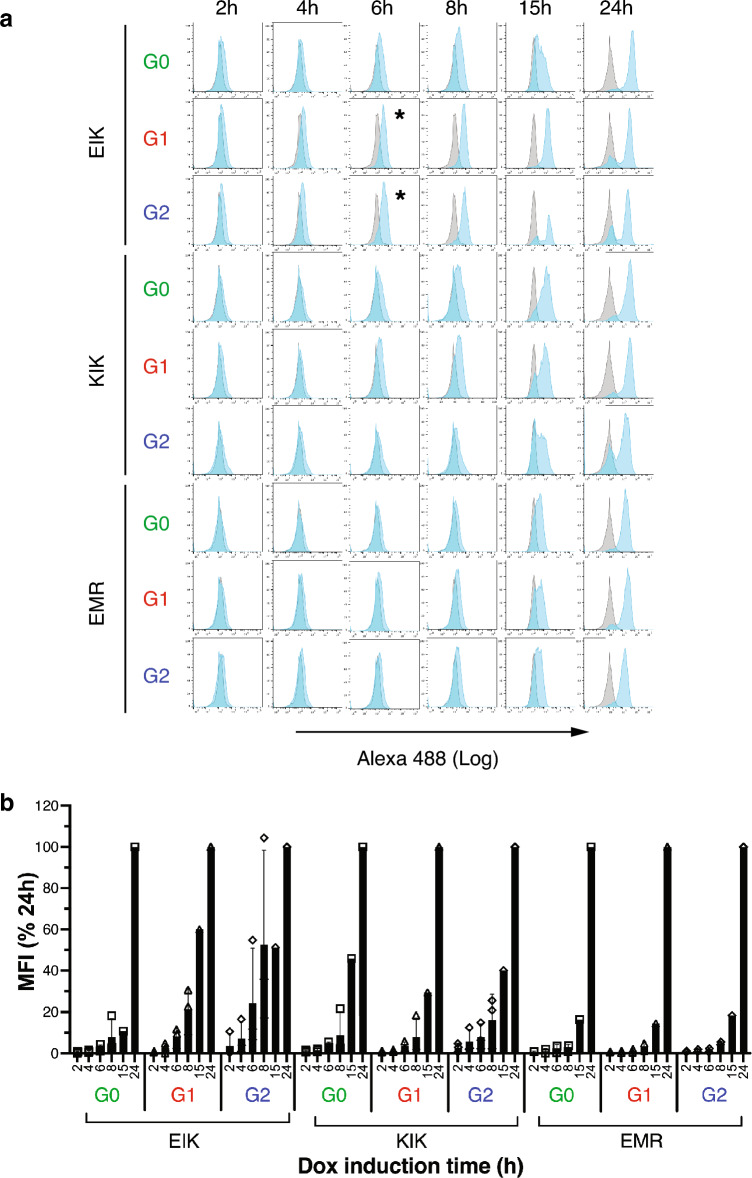
Time-course of APOL1 appearance on the podocyte surface. (**a**) iAPOL1 podocytes induced with 5ng/ml dox for the indicated times were subjected to FACS with Alexa488-conjugated 3.7D6 anti-APOL1 (cyan) and plotted on the same histogram as the uninduced control (grey). Data is representative of 2–3 experiments. The two toxic haplotypes (G1 and G2 EIK) start to shift a couple of hours earlier than the others (*), likely due to earlier and more facile expression (see Supplementary Fig. 9). (**b**) Mean fluorescence intensities (MFI) of APOL1 on iAPOL1 EIK (*n* = 3), KIK (*n* = 3) and EMR (*n* = 2) podocytes are plotted versus time as a percentage of that at the 24h timepoint to better illustrate the kinetic differences in surface arrival. Mean and SD of 2–3 experiments are plotted; open symbols show individual datapoints.

APOL1-G1 EIK reportedly traffics faster to the surface of HEK-293 cells than G2-EIK and G0-KIK^9^, so we investigated our iAPOL1 podocytes. APOL1-G1 and G2-EIK appeared at the cell surface at lower dox concentrations than G0-EIK (Fig. 3 and Supplementary Fig. 1), implying faster transport and/or more facile induction. To distinguish between these, we followed the time-courses of induction by Western blotting and plasma membrane arrival by FACS. Both APOL1-G1 and G2 EIK were faintly detectable by Western blotting as early as 4h after induction (Supplementary Fig. 9a) and started to appear at the cell surface 2h later, 6h post-induction (Fig. 4), increasing over time. G0-EIK took 2h longer both to be expressed and to traffic to the plasma membrane, implying slower induction. This was supported by the dose-dependence of expression: G0-EIK required 2–5 × more dox than G1 and G2-EIK (Supplementary Fig. 9b). In the KIK haplotype, all three variants arrived at the plasma membrane with similar kinetics to G0-EIK (6-8h) and the three EMR variants took 8-15h (Fig. 4). Thus, G1 and G2-EIK appear more readily induced rather than faster trafficked per se.

We next followed the kinetics of podocyte swelling, which precedes APOL1-mediated cytotoxicity^8,10,34^. Swelling was first evident around 8h for G2-EIK (Supplementary Fig. 10), a mere 2h after its arrival at the cell surface (Fig. 4). Despite similar trafficking kinetics, G1-EIK took longer to induce swelling, starting around 16h, suggesting that G2-EIK is more potent, in agreement with its higher cytotoxicity (Fig. 3a), as in HEK-293 cells^9^. As expected, no swelling was observed with G0-EIK or any other non-toxic haplotype in 24h (Supplementary Fig. 10 and data not shown). Thus, APOL1-G1 and G2-EIK arrival at the plasma membrane precedes podocyte swelling by at least 2h and precedes cytotoxicity by several hours, as in HEK-293 cells^9,10^.

### APOL1 must reach the plasma membrane to cause cell death.

**Figure 5:**
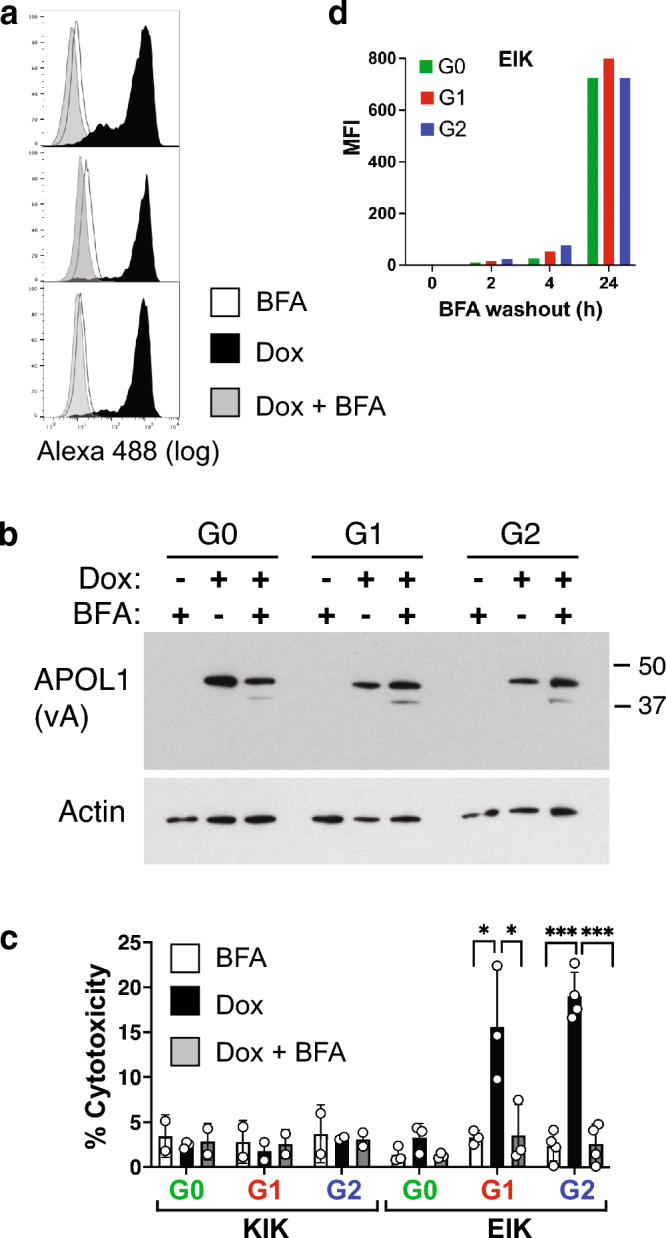
APOL1 must reach the plasma membrane to cause cell cytotoxicity. (**a**) Flow cytometry with anti-APOL1 3.6D12 of iAPOL1.vA-EIK podocytes treated with 5µg/ml BFA only (white), 25ng/ml Dox (black) or both (grey) for 24h. Data is representative of 3 experiments. (**b**) APOL1 Western Blot of 10µg lysates of the other half of the same samples as in (a) showing that BFA does not inhibit dox-induced APOL1 expression. Actin is the loading control. Full length blots are shown in Supplementary Fig. 23. (**c**) CytoTox-Glo™ of iAPOL1-EIK and KIK (vA) podocytes treated as in (a) for 24h. Bars are mean and SD of 2–4 duplicate experiments (circles are individual data points). BFA prevented G1/G2-EIK cytotoxicity, with no effect on the non-toxic G0-EIK or negative control KIK variants. *, *p* < 0.05; ***, *p* < 0.001. (**d**) iAPOL1.vA-EIK podocytes were treated with 25ng/ml Dox and 5µg/ml BFA for 24h, then washed, chased and subjected to FACS with 3.6D12 after different times of washout. Mean fluorescence intensities are plotted.

To determine if APOL1 arrival at the plasma membrane is required for cytotoxicity, we used the ER-Golgi transport inhibitor Brefeldin A (BFA) and verified that plasma membrane, but not total, APOL1-EIK expression was successfully abolished (Fig. 5a,b). BFA completely rescued dox-induced G1 and G2 cytotoxicity (Fig. 5c), confirming that APOL1 must reach the plasma membrane in podocytes, as previously shown in HEK-293 cells^9,14^. We next took advantage of BFA washout to compare ER to plasma membrane trafficking times of 24h-synthesized APOL1, when G0 expression had caught up with G1 and G2 (Supplementary Fig. 9a). All three EIK variants started arriving at the surface within 2h of washout, with G2 and G1 being only marginally faster than G0, given their similar total surface levels after 24h washout (Fig. 5d). This confirms that earlier induction contributes more to the faster G1 and G2-EIK arrival at the cell surface (Fig. 4) than does faster trafficking.

The G2-EIK podocytes started swelling within 1.5h of BFA washout, almost immediately after arriving at the surface (Supplementary Fig. 11), in line with the timings with the RUSH system in APOL1-HEK-293 cells^9^. G1-EIK was again less potent despite similar trafficking, only starting to swell at 3.5h washout, whereas barely any G0-EIK cells started swelling by 21.5h, by which time all the G1 and G2 cells were swollen (Supplementary Fig. 11b).

### Cytoplasmic APOL1-G1 and G2 isoforms vB3 and vC are not toxic to podocytes.

**Figure 6:**
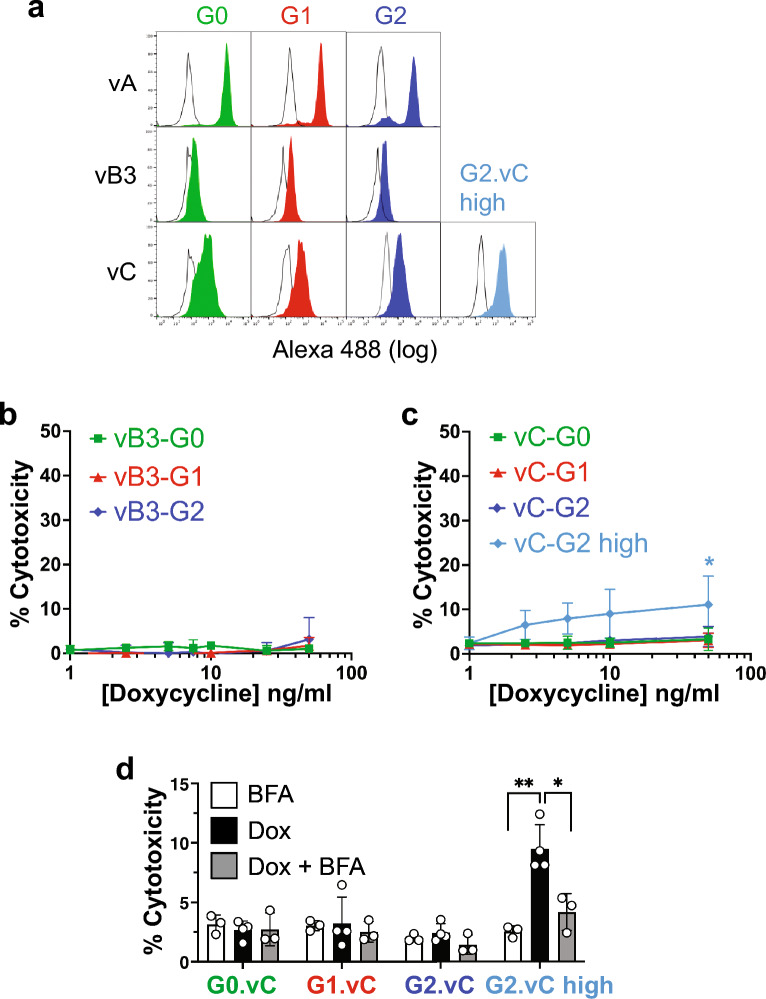
Cytoplasmic APOL1 isoforms are not cytotoxic. (**a**) FACS of dox-induced (16h 10ng/ml) APOL1.vB3 and vC-EIK podocytes with anti-APOL1 3.6D12, showing almost no surface expression of isoform vB3 and only a little expression of vC compared to secretory isoform vA variants, which were used throughout the rest of this work and re-run here for comparison. Two clones of G2.vC are shown: G2.vC expresses similar total levels to G2.vA (Supplementary Fig. 13b), while G2.vC-high (light blue) with more luminal APOL1 (Supplementary Fig. 12b) consequently has higher cell surface levels, albeit still well below those of G2.vA. (**b**) CytoTox-Glo™ assay of APOL1.vB3 (EIK) variants showing no cytotoxicity at 72h even up to 50ng/ml dox. Graph is plotted on the same scale as Fig. 3a for easier comparison with APOL1.vA-EIK (48h at ≤ 25ng/ml). Mean and SD of three independent duplicate experiments. (**c**) CytoTox-Glo™ assay of APOL1.vC (EIK) variants showing no cytotoxicity in 48h for clones expressing similar levels to APOL1.vA, even at 50ng/ml dox. The APOL1.vC-G2 high clone (light blue) exhibits minor cytotoxicity, although far less than APOL1.vA-G2 EIK (Fig. 3a). *, *p* < 0.05 vs no dox control. (d) Rescue of APOL1-G2.vC-high cytotoxicity by BFA. iAPOL1.vC-EIK podocytes were treated for 48h with 25 ng/ml dox (black), 5µg/ml BFA (white), or 48h dox with BFA added for the last 24h only (grey), because BFA was toxic after 48h. Bars are mean and SD of 3–4 experiments (circles are individual data points). *, *p* < 0.05; **, *p* < 0.01.

Since APOL1-EIK must reach the podocyte surface for cytotoxicity, we predicted that cytoplasmic isoforms of APOL1 lacking complete signal sequences (vB3, vC; Fig. 1a)^14,19,33^ would not kill podocytes, although APOL1 expressed in the cytoplasm via signal sequence removal *did* kill HEK-293 cells^35^. We therefore generated single cell clones of APOL1-G0, G1 and G2 isoforms vB3 and vC (all EIK, unlike Khatua et al.'s KIK HEK-293 transients)^33^. Like G0-KIK^19^, all three APOL1.vB3 EIK variants were on the cytoplasmic face of the ER, while vC exhibited mixed topology, with most, but not all, being cytoplasmic (Supplementary Fig. 12), in agreement with the topologies assessed in HEK-293 cells using a GlycoTag assay^35^. Accordingly, a slight FACS shift indicates minor surface expression for vC, and barely any for vB3 (Fig. 6a). Neither vB3 nor vC isoforms were toxic when G1 or G2 were expressed at similar total levels to vA (Fig. 6b,c and Supplementary Fig. 13). A higher expressing G2.vC clone with more luminal and surface expression did exhibit minor cytotoxicity (speculatively due to a minimally active signal sequence^19^ combined with overexpression), but this was diminished by BFA (Fig. 6c,d), indicating that it is the secretory, not cytoplasmic, fraction that is responsible for toxicity. This suggests the ability of signal sequence-free APOL1 to kill HEK-293 cells^35^ is either a peculiarity of that cell line or a transient expression artifact^19^. Our isoform data independently support the requirement for surface expression for APOL1-mediated toxicity in podocytes.

### APOL1-G1 and G2-EIK cluster more readily on the podocyte surface.

**Figure 7:**
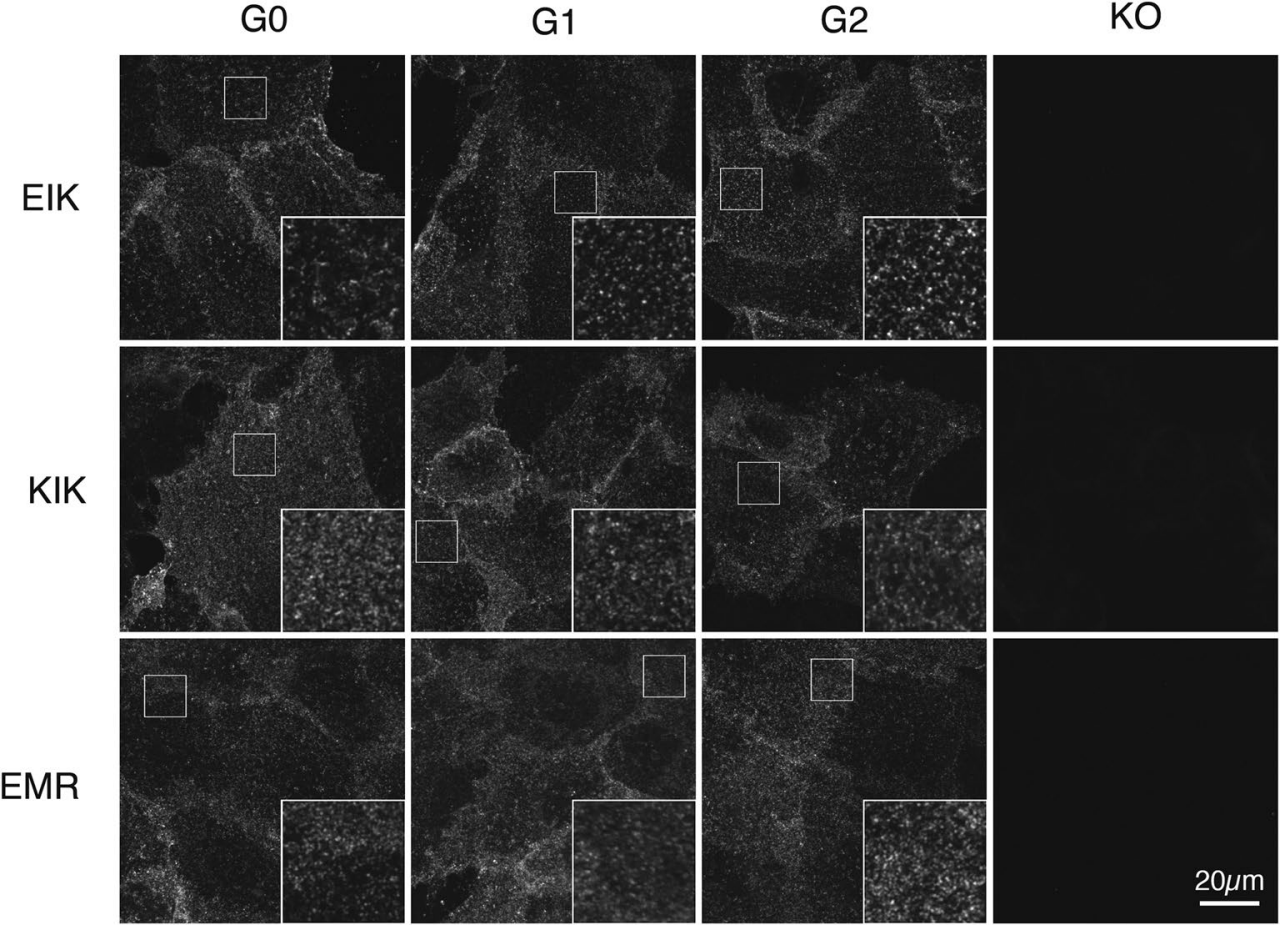
APOL1-EIK variants form more discrete clusters on the podocyte surface than KIK or EMR. iAPOL1-G0, G1, G2 or KO (uninduced G2) podocytes of EIK (upper), KIK (middle) and EMR (lower) haplotypes were incubated for 1h on ice with 5µg/ml 3.6D12 (1.24% aggregated), then warmed for 15 min to induce any internalization. Cells were then washed, fixed, Triton permeabilized and stained with Cy3-anti-mouse. Insets are 3 × magnification of the boxed region. Data are representative of two experiments with this antibody (see Supplemental Figs. 14, 16 for other antibodies).

As plasma membrane expression is clearly essential for cytotoxicity, we examined whether G1 and G2-EIK were differently localized on the plasma membrane to the others. Live podocytes were incubated on ice (to avoid internalization) with monoclonal anti-APOL1 antibodies to different epitopes that detect native APOL1 well by FACS^32^, then washed, fixed, permeabilized and detected with fluorescent secondary antibodies. Interestingly, with four of the antibodies, including 3.6D12 (Fig. 7) and 4.2C4 (Supplementary Fig. 14), APOL1-G1-EIK and G2-EIK virtually all congregated into large clusters, whereas G0-EIK exhibited fewer large clusters with more “smooth” APOL1 staining in between. All three KIK and EMR variants exhibited more homogeneous staining than G0-EIK, suggesting they are less prone to clustering than EIK.

By contrast, two highly aggregated antibodies (5.17H8 and 5.17G5, with 16% and 50% aggregation, respectively) caused clustering of all nine haplotypes (Supplementary Fig. 15a), suggesting antibody-mediated crosslinking was responsible. Clustering was indeed mediated by aggregation (≥ 0.7%) rather than epitope because more stringently purified batches of 3.6D12 and 5.17H8 (as well as 5.17D12) with 0% aggregation yielded smooth staining even on G2-EIK (Supplementary Figs. 15b, 16), and addition of a secondary antibody prior to fixation cross-linked them into clusters like the highly aggregated antibodies (Supplementary Fig. 17). Two commonly used commercial anti-APOL1 polyclonals similarly caused prounounced clustering (Supplemental Fig. 16). Clustering was not Fc-receptor mediated because all our recombinant APOL1 antibodies are effectorless (LALAPG)^36^. None of the puncta of any haplotype or size colocalized with either clathrin or G_M1_ (Supplementary Figs. 18–21), even after warming for 5 or 15 min, respectively, to induce internalization, suggesting they are not clathrin-coated vesicles or lipid rafts (including caveolae), consistent with lack of rapid internalization^37^. We therefore interpret this antibody-mediated clustering data as a greater propensity for G1 and G2-EIK variants (and to a lesser degree G0-EIK) to oligomerize on the cell surface than the non-toxic haplotypes, perhaps reflecting more facile ion channel formation capabilities, although we were unable to find a suitable method to quantitate this satisfactorily. The clusters are larger than individual channels, however, appearing similar in size to clathrin-coated vesicles (~ 100nm; Supplementary Figs. 18–20).

**Figure 8:**
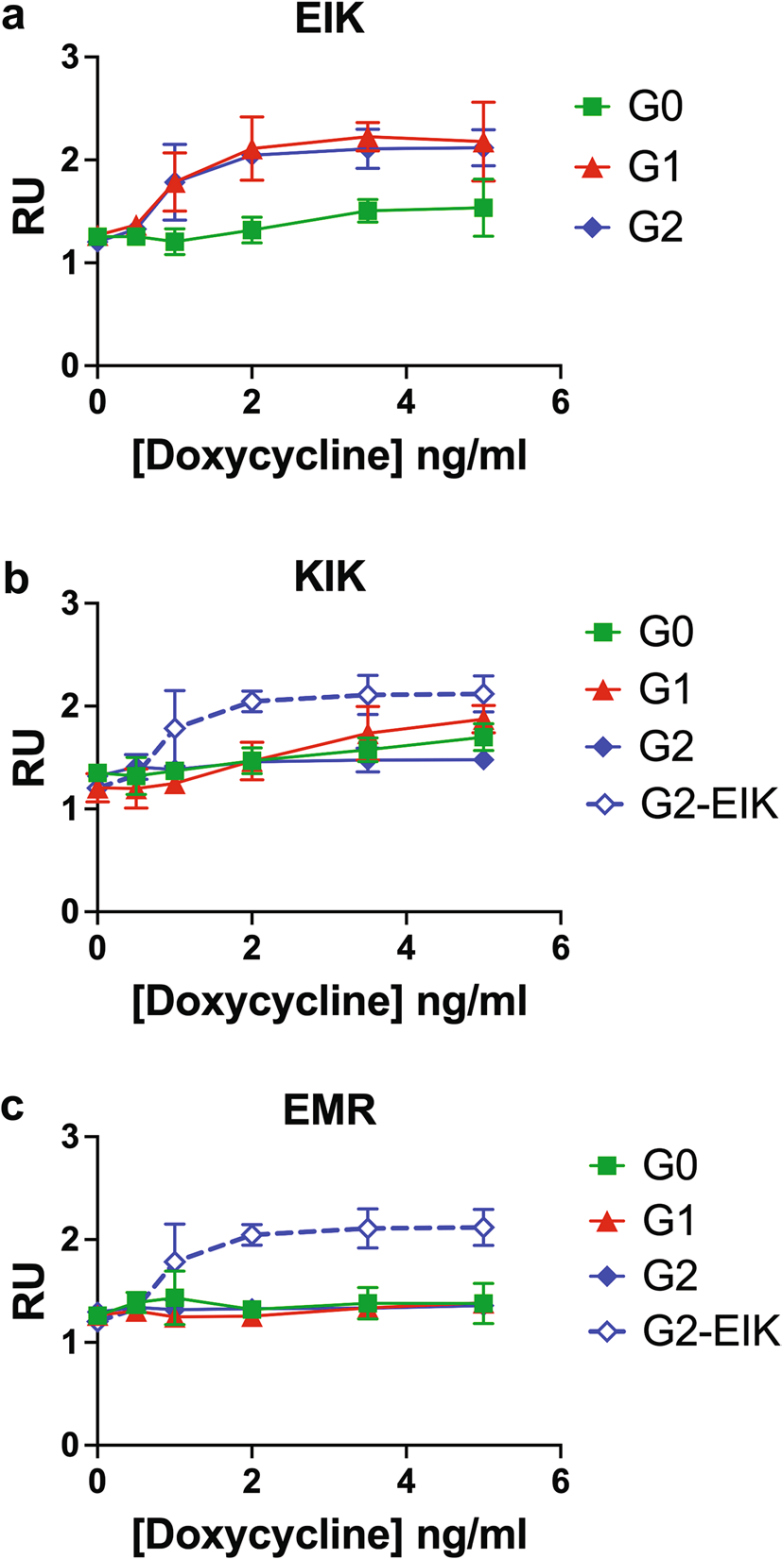
APOL1-G1 and G2-EIK conduct more K^+^ ions than the non-toxic haplotypes. iAPOL1 podocytes were incubated overnight with 0-5ng/ml dox, and subjected to thallium FLIPR as a proxy for K^+^ conductance. (**a**) G1 (red filled triangle) and G2 (blue filled diamond) EIK conduct more than G0-EIK (green filled square), the three KIKs conduct little (**b**) and the EMRs not at all (**c**). G2-EIK (blue diamond) is included as a comparator in b and c. Means and SDs of 3 (EMR) or 4 (EIK, KIK) independent duplicate experiments are plotted at the 60 s timepoint. RU, Relative Units.

If APOL1 kills podocytes via cation channel activity, then G1 and G2-EIK should conduct more cations. Using the thallium FLIPR assay^38^ as a proxy for K^+^ currents, we indeed measured higher APOL1-dependent currents for G1 and G2-EIK than G0-EIK and the other haplotypes (Fig. 8). The baseline conductance (of endogenous K^+^ channels) was similar in all uninduced haplotypes, but increased with dox concentration in G1 and G2-EIK, supporting its dependence on APOL1 induction level. There were much smaller currents in the KIK and G0-EIK clones and no current at all above background in the completely non-toxic EMR clones. Unexpectedly, G2-EIK did not conduct more than G1-EIK, but its channel activity may have been confounded by the earlier onset of cytotoxicity (Supplementary Figs. 2a, 10). Thus, cation conductance in podocytes correlates with cytotoxicity, as anticipated, as well as with apparent propensity for surface clustering.

## Discussion

We demonstrate that APOL1-mediated cytotoxicity in an immortalized podocyte cell line is dose-dependent, requires transport to the cell surface, and is limited to the G1 and G2 risk variants in their most common “African” EIK haplotype background, with much slower killing by G0-EIK and almost none by the three KIKs. While we refer to the EIK haplotype as “African” for simplicity due to its majority in that population, EIK is also found in a small percentage of Hispanics^9^ and in African diaspora, including admixed African Americans and Caribbeans. G1 and G2-EIK show identical localization and topology to the non-toxic haplotypes, with the exception of having a greater propensity for antibody-mediated clustering on the podocyte surface, perhaps reflecting a greater ability to oligomerize into functional cation channels and initiate the cascade of events leading to cytotoxicity^8^.

Our data extend previous results in HEK-293 cells^8,9,13–15^ in a more physiologically relevant cell type and clarify several issues. First, it suggests that there are cell-type differences when overexpressing APOL1 to model AMKD with respect to much diminished or no cytotoxicity of G0-EIK, and all KIK and EMR variants in podocytes versus HEK-293 cells. Second, it highlights the need for reporting *all* sequence variations of APOL1 used in any study^15^ (noting Origene NM_003661 clone marketed as G0-EMR is actually G0-KIK). Third, for comparing APOL1 risk variants mechanistically, matching the haplotypes (ideally using African EIK for all) is important, since EIK is more toxic than KIK. Several labs have been using G0-KIK instead of G0-EIK as a control for G1/G2-EIK, justifying G0-KIK as being the native “AfCom” haplotype^9,10,15,16^, whereas we now show G0-EIK is in fact up to 3 × more prevalent in Africans; consequentially G0-KIK should no longer be called “AfCom”. Fourth, our identification of G2-KIK, G1-EMR and G2-EMR human samples implies that these could account for a small fraction (up to ~ 6%) of the ~ 80% African Americans with risk G1/G2 who do not develop AMKD, although their low frequencies may not justify genotyping in the clinic. Fifth, since only surface APOL1 is active, it is better to select APOL1-expressing clones by equal surface (FACS) rather than total (Western blot) expression to compare activity. Sixth, we clarify that faster arrival of G1/G2-EIK at the cell surface in our clones is due to faster expression more than trafficking differences. This was somewhat surprising to us, since previous ER stress data^11^ led us to expect G1 and G2 would be more misfolded than G0, fail ER quality control and hence reach the surface more slowly.

That plasma membrane localization is required for APOL1 cytotoxicity agrees with earlier studies in HEK-293 cells^9,14^ and supports the notion that secretory APOL1 risk variants (the major vA isoform in podocytes)^19^ are responsible for AMKD. Our isoform topology data matches the GlycoTag results of Müller et al^35^, with vA and a minority of vC being luminal, while vB3 was fully cytoplasmic and non-cytotoxic. Although APOL1-G2.vB3 enhanced podocyte loss in a uninephrectomy model^39^, this was most likely via IL-1 β secretion and inflammation rather than ion channel activity. Our ability to rescue APOL1.vC cytotoxicity with BFA indicates the secretory, not cytoplasmic, portion causes cytotoxicity. We speculate that APOL1.vC cytotoxicity (without BFA) may be an overexpression artifact, as it only occurred in the highest expressing clone and endogenous vC in untransfected podocytes is expressed ~ 30 × lower than vA. Our data thus support the proposal that splice-switching of vA to vC (by skipping exon 4) could potentially be therapeutic^40^.

The data that APOL1-G1 and G2-EIK act as cation channels to mediate cytotoxicity is now very strong. Podocyte swelling occurs within minutes of G2-EIK arrival at the plasma membrane following BFA washout, supporting ion channel activity rather than later dysregulation of mitochondria or endosomal maturation defects^41^ as the key initiating event. APOL1-G1 and G2-EIK import cations across the plasma membrane^9^ and contribute to K^+^ efflux (Fig. 8 and refs^5,8,17^). K^+^ efflux from APOL1-containing liposomes mirrors our cytotoxicity and FLIPR data, with each EIK variant conducting more than its KIK counterpart, followed by its EMR counterpart; and G1/G2-EIK conducting more than G0-EIK^7^. Patch-clamp studies in HEK-293 cells also agree with our data: O’Toole et al. found no differential current between their three EMR variants with no differential cytotoxicity (although they did have cytotoxicity)^13^; and during preparation of this manuscript Vandorpe et al. showed that APOL1-G2 EIK and G1-EIK conduct more than G0-KIK (G0-EIK was not tested)^16^. Our dataset is the only one to compare all stably transfected haplotypes in a single experiment and suggests cation flux triggers cytotoxicity. Most compellingly, the almost 50% amelioration of proteinuria in FSGS patients by VX-147 overwhelmingly supports cation channel activity as the major role of APOL1 in AMKD^5^, assuming it prolongs kidney function in the ongoing placebo-controlled phase II/III study (NCT05312879).

Exactly how the EIK haplotype enables the G1 and G2 risk variants to conduct more cations is unclear. We ruled out differences in localization or topology, and attributed slightly faster plasma membrane arrival to faster translation over faster secretory transport. Although the structure of G1 and G2 (EMR) RNAs reportedly differ to G0’s, they *decreased* global translation via PKR binding and eIF2a phosphorylation^42^, so do not explain our observations. Our translational differences are likely just clonal due to insertion at different PiggyBac (TTAA) sites, preferentially located in transcriptional units^43^, since we picked the highest expressing clones. In any case, a couple of hours head-start over G0 arrival at the surface is unlikely to explain the differential conductance after 16 h. We therefore favor our interpretation that the greater antibody-mediated clustering on the cell surface by G1/G2 EIK could reflect a greater propensity for oligomerization of EIK channels into an active (or activatable) state, although our evidence is indirect and differential oligomerization needs to be validated biochemically and quantitatively. G1 and G2 of all haplotypes appear to insert better into membranes at acidic pH^7^, so a greater amount might be productively inserted during transit through the acidic Golgi complex^9,44^. This is supported by Raper and colleagues’ finding that transient acidification of APOL1-HEK-293 cells increases the cytotoxicity of all three variants^9^. Furthermore, they showed APOL1 dimerization within the membrane was required for channel activity in vitro, mediated by the leucine zipper in the SRA-ID^20^, near G1 I384M and the G2 deletion. While more N-terminal, E150 in the N-terminal domain and K255 near the 3rd transmembrane domain are both predicted to be extracellular like the leucine zipper^20,32^ and could conceivably influence oligomerization either directly or perhaps via binding another protein. I228 is at the end of the 2nd putative transmembrane domain, which forms part of the helix-loop-helix thought to be important for anchoring APOL1 in the membrane^45^, so its substitution for M228 might somehow affect the pool of APOL1 available for interactions within the membrane. An atomic structure of full length APOL1 would be greatly informative in this respect.

Limitations of our study include that we used undifferentiated immortal podocytes because differentiation resulted in greater variability in our cytotoxicity assays, although the overall cytotoxicity trends were recapitulated, suggesting this was not a big issue (data not shown). Additionally, the high surface expressers we selected may not reflect APOL1 levels or localization in AMKD, as there is only limited data on APOL1 plasma membrane staining in human kidneys^19,21^. Nonetheless, we saw cytotoxicity at 1ng/ml dox, which we have shown induces equivalent APOL1 expression to interferon gamma-stimulated wild type podocytes^32^, which in turn express ~ 4 × more than *non*-inflamed normal human kidneys^19^. Another limitation is that some of the differences between haplotypes could be due to clonal variation, although we attempted to mitigate this by reproducing the results with two other clones. Furthermore, our cytotoxicity trends largely agree with Lannon et al.^15^ with the exception that HEK-293 cells appear more sensitive to APOL1 cytotoxicity overall, and in particular to G1-KIK and G2-KIK, which kill HEK-293 cells almost as well as their EIK counterparts (see Fig. S5 of Lannon et al.)^15^. We speculate the sensitivity of HEK-293 cells to G0-EIK and KIKs may be because they don’t normally express APOL1, so may not express a hypothetical G0-binding protein postulated by Limou et al.^26^ to limit its activity; furthermore, we hypothesize KIK may facilitate binding to such a protein. Indeed, lack of G0 dominance has been demonstrated in HEK-293 cells^10^ as well as APOL1-Tg mice^46^, a species normally lacking APOL1, so it will be important to test for dominance in our human iAPOL1-podocytes, since G0 gene therapy could potentially rescue AMKD. It will also be of interest to determine whether endothelial cells, the other major APOL1-positive cell type in kidneys^19,47,48^, are killed by EIK but not KIK risk variants to better understand why KIK appears protective in some cell types.

In summary, we propose that G1 and G2 EIK channels are more active than G0, due to more facile oligomerization into channels and/or greater propensity to open or conduct through the cation pore. Quantitative evaluation would strengthen this conclusion. It has been proposed that E150K SNP associates with protection from end stage renal disease (OR 0.77)^25^ and FSGS (OR 0.42)^49^ in African Americans, although strong differences in risk allele distribution across the ancestral haplotypes could have led to spurious protective signals. Our in vitro data support the possibility of protection by E150K (KIK) at least in podocytes. Hence K150E may be key for mediating disease in the context of EIK but not EMR. Our identification of G1/G2 in EMR and KIK backgrounds suggests this would be worth investigating from a human genetics standpoint; however, as our clinical samples did not have information on kidney disease, we could not test this. If protection by KIK holds true, then since 95–98% of G1 and G2 are on the EIK haplotype in Africans (Supplementary Table S2), this suggests CRISPR of E150 to K150 could be a potential one-time therapy for AMKD of all combinations of EIK risk variants once this technology is ready for kidney delivery. This would offer an advantage over small molecule ion channel inhibitors in that it would not inhibit APOL1-mediated immunity against trypanosomes.

## Methods

### Cell lines and constructs

We previously used human immortal AB8/13 podocytes (under licence from Prof. Moin Saleem, University of Bristol) to knock out endogenous APOL1 and stably retransfected APOL1-G0, G1 and G2 KIK under a doxycycline (dox)-inducible promoter to create i*APOL*1-podocytes (“i” stands for inducible), as published^19,32^. Herein we used the same methods to generate the other haplotypes. Specifically, to generate the “EIK” haplotypes, plasmids encoding the G0, G1 and G2 KIK variants (all secretory isoform vA) were subjected to site directed mutagenesis using primers GGTTGAAAAGTgAGCTTGAGGATAAC and GTTATCCTCAAGCTcACTTTTCAACC (lower case indicates the base pair change directing the K150E mutation). Subcloning of a PshAI-NotI C-terminal fragment was used to convert G0-KIK isoforms of APOL1.vB3 and vC into EIK haplotypes. APOL1-G0 EMR was synthesized at GenScript. To create G1 and G2 variants, the G0 C-termini were swapped for G1 or G2 by cutting and pasting with SphI or AccI, respectively. All were subcloned into our custom dox-inducible PiggyBac vector^32^ with AgeI and SalI and the inserts were fully verified by DNA sequencing. APOL1-KO podocytes (clone 89401–3) were transfected and up to 10 clones of each variant in each haplotype were obtained by limiting dilution from individual cells to create single cell clones as before^32^. Due to a liquid nitrogen shortage during the pandemic, clones were stored at − 80 °C, which led to loss of viability and expression in some cases (e.g. G2-KIK) in later experiments.

As before^19,32^, podocytes were grown at the permissive temperature of 33 °C in growth media consisting of RPMI, 10% tetracycline-free FBS (Takara 631101), 1% insulin/transferrin/selenium (Gibco 41400–045), 1% glutamine, 1% penicillin/streptomycin and 5µg/ml puromycin.

### Podocyte toxicity assay

6 × 10^4^ cells (for 48h induction) were plated in 96-well black wall clear bottom plates (Costar 12–566-70). 24h later, media was replaced with 50 µl of fresh media containing 0–50 ng/ml doxycycline (Alfa Aesar J60422). At 24-120h post induction (typically 48h), cytotoxicity was measured using the CytoTox-Glo™ assay (Promega G9290), which measures extracellular protease activity released from dead cells followed by total cell number after digitonin permeabilization. Luminescence was recorded using the Spectra Max instrument software v6.5. Percentage of cell death was calculated by normalizing pre-digitonin signal to post-digitonin signal to obviate any differences in growth rate. Cell death due to dox treatment was calculated by subtracting background no dox values. Data was analyzed in Excel, and plotted using GraphPad Prism 9.5.1. The Student’s unpaired *t*-test was used to compare cytoxicity of the variants to the KO control unless otherwise indicated.

### Brefeldin A

Cells at 70% confluency in growth medium were treated with 25ng/ml dox with or without 5µg/ml Brefeldin A (Biolegend 420601) for 17h.

### FLIPR® potassium channel assay

Podocytes seeded at 3000 cell/well in 384-well plates were induced with different concentrations of dox for 16 h, then subjected to K^+^ thallium FLIPR assay (Molecular Devices R3622C) according to the manufacturer’s instructions. Briefly, cells were loaded with 1 × component A dye in HBSS at room temperature for 1 h**.** Then the plate was transferred to a FDSS/μCELL machine (Hamamatsu C13299) to start continuous recording fluorescence signal at excitation wavelength 485 nm and emission wavelength 525 nm at 1 Hz. Baseline reading in the presence of compound A was recorded for 15 s, then 10 mM thallium was added and recorded for 5 min. The signal fold change between 60 s and baseline was plotted vs dox concentration in GraphPad Prism v9.5.1.

### Flow cytometry (FACS)

FACS was performed as previously with 2.5 µg/ml anti-APOL1 mouse monoclonals^32^ and 2 µg/ml Alexa488 anti-mouse (Invitrogen A11029); or with 2.5 µg/ml of Alexa-488 conjugated 3.7D6, labeled according to the manufacturer’s instructions (Molecular Probes A10235) to achieve 4.2 dye/antibody. Signals were read in a FACS Calibur or FACS Celesta (Becton Dickinson), and live cell (propidium-iodide negative) data was analyzed using FlowJo v8.4.5 or v10.8.1 and the *y*-axis was normalized to mode. Plotted measurements of mean fluorescence intensities (MFIs) included the entire distribution of APOL1 expression.

### Western Blotting

Total expression levels were determined by Western blotting as earlier^32^ on 4 or 10µg native RIPA buffer lysates. A mixture of 0.05µg/ml anti-APOL1 rabbit monoclonals 3.7D6 and 3.1C1^19^, followed by HRP anti-rabbit (Jackson 711–036-152, 1:8000) was used for detection. After stripping (Pierce Restore Plus), HRP-conjugated anti-actin (13E5, Cell Signaling Technologies 5125S at 1:5000) or rabmab anti-GAPDH EPR16891 (0.93µg/ml Ab181602, lot GR217575-61) followed by HRP anti-rabbit were used as loading controls.

### Immunofluorescence microscopy

Podocytes were fixed in 3% paraformaldehyde and permeabilized for 4–5 min with either 0.1% Triton-X-100, 0.0625% (62.5µg/ml) digitonin (Sigma D141) or for 20 min with 0.4% saponin, then stained and imaged by confocal spinning disk microscopy as before^19^.

Specifically for Supplementary Figs. 4 and 5, cells were PFA fixed, permeabilized with Triton and triple stained with antibodies to APOL1 (3µg/ml rat 4.17A5^19^), detected with 1.88µg/ml Cy3-anti-rat (Jackson 712–166-153); rabbit anti-calnexin cytoplasmic domain (an ER marker, green in Supplemental Fig. 4; 0.25µg/ml (Ab22595 Lot GR321610-1)) followed by Alexa647 anti-rabbit (Jackson 711–606-152); and cytochrome C (green in Supplementary Fig. 5), detected with 1.88µg/ml Alexa488 anti-mouse cross-adsorbed against rat (Jackson 715–546-151).

For Supplementary Fig. 6, APOL1 was detected in digitonin-permeabilized podocytes with a murine monoclonal antibody to its N-terminal domain (2µg/ml 4.17A5; IgG2a^19,32^) followed by 2µg/ml Alexa488-anti-mouse IgG2a (Molecular Probes A21131). Anti-calnexin extracellular (luminal) domain clone 37 (1:100 mouse IgG1, Becton–Dickinson 610524) was detected with 2µg/ml Alexa647-anti-mouse IgG1 (Molecular Probes A21240). Nuclei were stained with DAPI in Prolong™ Gold mounting medium (Invitrogen P36931). Confocal spinning disk microscopy was with a 63 × objective (1024 × 1024, bin 1) as previously published^19^, except images were assembled with Adobe Photoshop 2022. Gamma changes were applied to the whole image.

### APOL1 clustering immunofluorescence

For surface APOL1 labeling, 5µg/ml anti-APOL1 antibodies^19,32^ (aggregation status determined by light scattering), as well as Proteintech 11486–2-AP and Sigma HPA018885 (lot E105900), were incubated with live podocytes on ice for 1h in complete carbonate-free media (Gibco 18045–088), washed 4x, 3% PFA fixed, saponin-permeabilized and detected with 1.88µg/ml Cy3-anti-mouse (Jackson 115–165-072), Alexa488 anti-mouse (Jackson 715–546-150) or Alexa488 anti-rabbit (Jackson 711–546-152). Small scale recombinant screening anti-APOL1 antibodies used in the clustering experiments were only protein-A purified, resulting in different extents of aggregation; larger scale preparations were further purified by size exclusion chromatography and mostly non-aggregated. For secondary antibody-mediated crosslinking, 5µg/ml rabbit anti-APOL1 was incubated for 30 min on ice, washed, then 1µg/ml Alexa488 anti-rabbit was added for 30 min on ice and the excess washed off prior to fixation. Lipid rafts were stained with the Vybrant™ Alexa-488 kit (Molecular Probes V34403) according to the manufacturer’s instructions with cholera toxin B crosslinking at 37 °C for 15 min. Clathrin was detected with 3µg/ml monoclonal X22 (ThermoFisher MA1-065) or 1:1500 rabbit polyclonal (Abcam Ab21679) in cells re-warmed for 5 min before fixation.

### Immunoelectron microscopy

Immuno-electron microscopy was performed on ultrathin cryosections as previously published^19^. In short, podocytes induced for 8h with 5ng/ml dox were fixed in 4% PFA in 0.1 M Sorensen Phosphate Buffer and stored in 0.6% PFA until preparation of cryosections at − 120 °C on cryo-ultramicrotomes Leica EM UC6 and UC7 (Leica Microsystems). Thawed cryosections were incubated with 1.9 µg/ml 3.6E10 anti-APOL1 (murine IgG2a), and directly detected with 10nm protein A-Gold (Cell Microscopy Core, University Medical Center Utrecht, The Netherlands) without a bridging step.

### Incuyte® Imaging

6 × 10^4^ cells were plated in clear bottom black wall 96-well plates (Costar 12–566-70) in growth medium. The next day, 5 ng/ml dox was added and plates were immediately imaged every 30 min at 33 °C using IncuCyte® Software (4 fields/well). Images were assembled in Adobe Photoshop 2022.

### Data mining methods

APOL1 G0, G1 and G2 variants were extracted from 1000 Genomes Project Phase 3 unrelated samples (see list at https://www.internationalgenome.org/data-portal/data-collection/30x-grch38) in the AFR, AMR, EAS and EUR super-populations (*n* = 2,012). The Genentech (GNE) cohort consisted of 32,716 patients from either internal clinical trials or external collaborations from the following disease areas: Alzheimer's, age-related macular degeneration, asthma, autoimmune disease, cancer, Crohn's disease, chronic obstructive pulmonary disease, inflammatory bowel disease, interstitial lung disease, idiopathic pulmonary fibrosis, multiple sclerosis, Parkinson’s disease, rheumatoid arthritis, systemic lupus erythematosus and ulcerative colitis, but not kidney disease. All GNE samples passed standard GWAS QC (relatedness checks, ancestry outliers, and excess heterozygosity). ADMIXTURE software was used to estimate the genetic ancestry of the GNE cohort using reference data from the 1000 Genomes Project and GNE samples with admixture rates >  = 0.7 in one of the 4 super-populations were included in this analysis. Beagle v5.4 was used to phase the APOL1 haplotypes in the GNE cohort using the variants shown in Fig. 1c. Haplotypes other than KIK and EMR with a frequency less than 1% were collapsed into one category.

### Supplementary Information

Supplementary Figures.

Supplementary Table S1.

Supplementary Table S2.

## Data Availability

The iAPOL1 podocytes and antibodies used in this study may be requested under MTA with Genentech, with the exception of 16% aggregated 5.17H8 (since repeat preps failed to aggregate as highly). For the APOL1 haplotype frequencies, the 1000 Genomes phase 3 public data is available at https://www.internationalgenome.org/category/phase-3/. However, the signed research consents from the patients permitted analysis of the whole genome sequencing data, but not publication of the raw data, hence we are not able to place that in a repository.
