# Supplementary material for: Apolipoprotein L1 (APOL1) renal risk variant-mediated podocyte cytotoxicity depends on African haplotype and surface expression
Source: Sci Rep. 2024 Feb 14;14:3765. doi: 10.1038/s41598-024-53298-4 (PMC10866943; doi:10.1038/s41598-024-53298-4)
Supplement: Supplementary file 1 — Supplementary Figures. [file 41598_2024_53298_MOESM1_ESM.pdf]

Apolipoprotein L1 (APOL1) renal risk variant-mediated podocyte cytotoxicity depends on African haplotype and surface expression.

Nidhi Gupta, Bridget Waas, Daniel Austin, Ann M. De Mazière, Pekka Kujala, Amy D. Stockwell, Tianbo Li, Brian L. Yaspan, Judith Klumperman and Suzie J. Scales

SUPPLEMENTARY MATERIAL INDEX

Figure S1. Histograms for dox dose response of APOL1 surface expression.
Figure S2. Time-courses and 72h dose responses of the different haplotypes of iAPOL1-podocytes by CytoTox-Glo^{TM}.
Figure S3. APOL1 cytotoxicity trends are reproduced in independent clones with similar expression, supporting haplotype-specific effects rather than clonal artifacts.
Figure S4. All APOL1 haplotypes localize to the ER.
Figure S5. None of the APOL1 haplotypes colocalize with mitochondria.
Figure S6. All APOL1 haplotypes have the same topology in the lumen of the ER.
Figure S7. Immunoelectron microscopy confirms no differences between APOL1 G0, G1 and G2 EIK localization.
Figure S8. All haplotypes exhibit similar surface topology by flow cytometry.
Figure S9. APOL1-G1 and G2-EIK express more readily than G0.
Figure S10. Podocyte cell swelling occurs at least 2h after arrival of APOL1-EIK at the cell surface.
Figure S11. iAPOL1-EIK podocyte swelling time-course after BFA washout.
Figure S12. Topology of APOL1 isoforms vB3 and vC by immunofluorescence.
Figure S13. APOL1 Western Blots of different isoforms showing total expression levels.
Figure S14. APOL1-EIK clusters more completely on the podocyte surface than KIK or EMR with a different APOL1 antibody.
Figure S15. APOL1 surface clusters are more pronounced with highly aggregated anti-APOL1 monoclonal 5.17H8.
Figure S16. APOL1 surface clustering correlates with aggregation, not epitope.
Figure S17. Secondary antibody-mediated clustering of monomeric anti-APOL1.
Figure S18. APOL1-G2 large (5.17H8-mediated) clusters are not in clathrin-coated pits.
Figure S19. APOL1-EIK large (5.17H8-mediated) clusters are not in clathrin-coated pits.
Figure S20. Smooth APOL1 and small APOL1 clusters are not in clathrin-coated pits.
Figure S21. APOL1 clusters are not in lipid rafts stained for GM1.
Figure S22. Full length APOL1/GAPDH Western blots for Figure 2b (all haplotypes).
Figure S23. Full length APOL1/actin Western blots for Figure 5b (BFA-treated iAPOL1.vA podocytes).
Figure S24. Full length APOL1/actin blots for Supplementary Fig. S9a (dox time course).
Figure S25. Full length APOL1/actin blots for Supplementary Fig. S9b (dox dose response).
Figure S26. Full length APOL1 Western Blots for Supplementary Fig. 13a (iAPOL1.vB3 podocytes).
Figure S27. Full length APOL1 Western Blots for Supplementary Fig. 13b (iAPOL1.vC podocytes).

Separately uploaded:

Table S1. Complete amino acid sequences of the APOL1 proteins expressed in this study (docx file).
Table S2. APOL1 haplotypes in 1000 Genomes and clinical data sets (xlsx file).

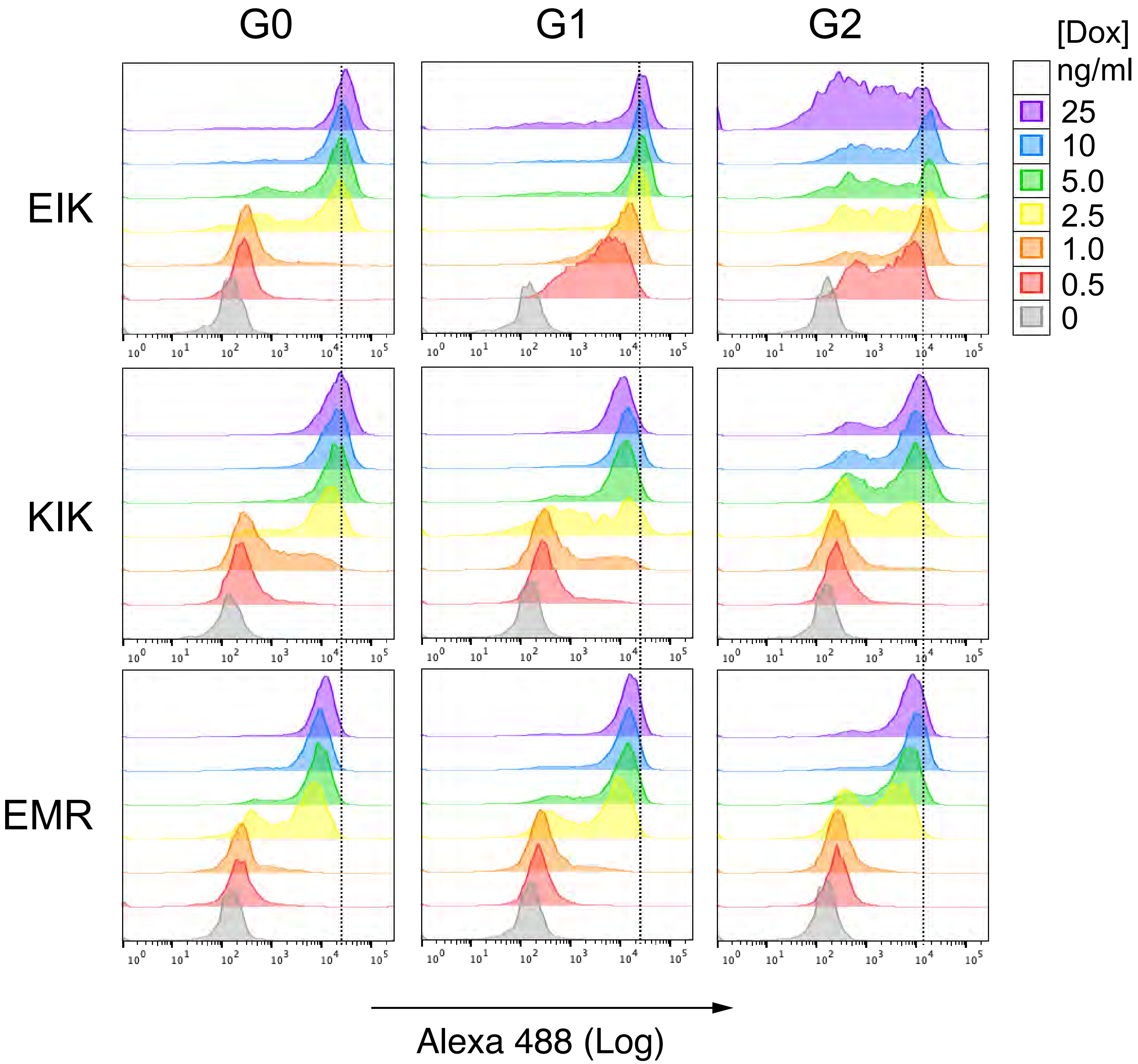

Figure S1. Histograms for dox dose response of APOL1 surface expression.
Representative FACS histograms of dox dose response in all nine iAPOL1 podocytes showing the G1 and G2 EIK variants start expressing at lower dox concentrations than G0-EIK and the other haplotypes after 16h induction (instead of the 48h used for cytotoxicity, to ensure sufficient cells remained alive to perform FACS with). Grey is uninduced, and colors in rainbow order indicate increasing dox concentrations from 0.5 to 25ng/ml. APOL1 was detected with 2.5µg/ml 3.6D12 (Ref. 32) followed by 2µg/ml Alexa488 anti-mouse, so is amplified compared to the direct staining with Alexa-conjugated primary antibody in Figs. 2 and 3.

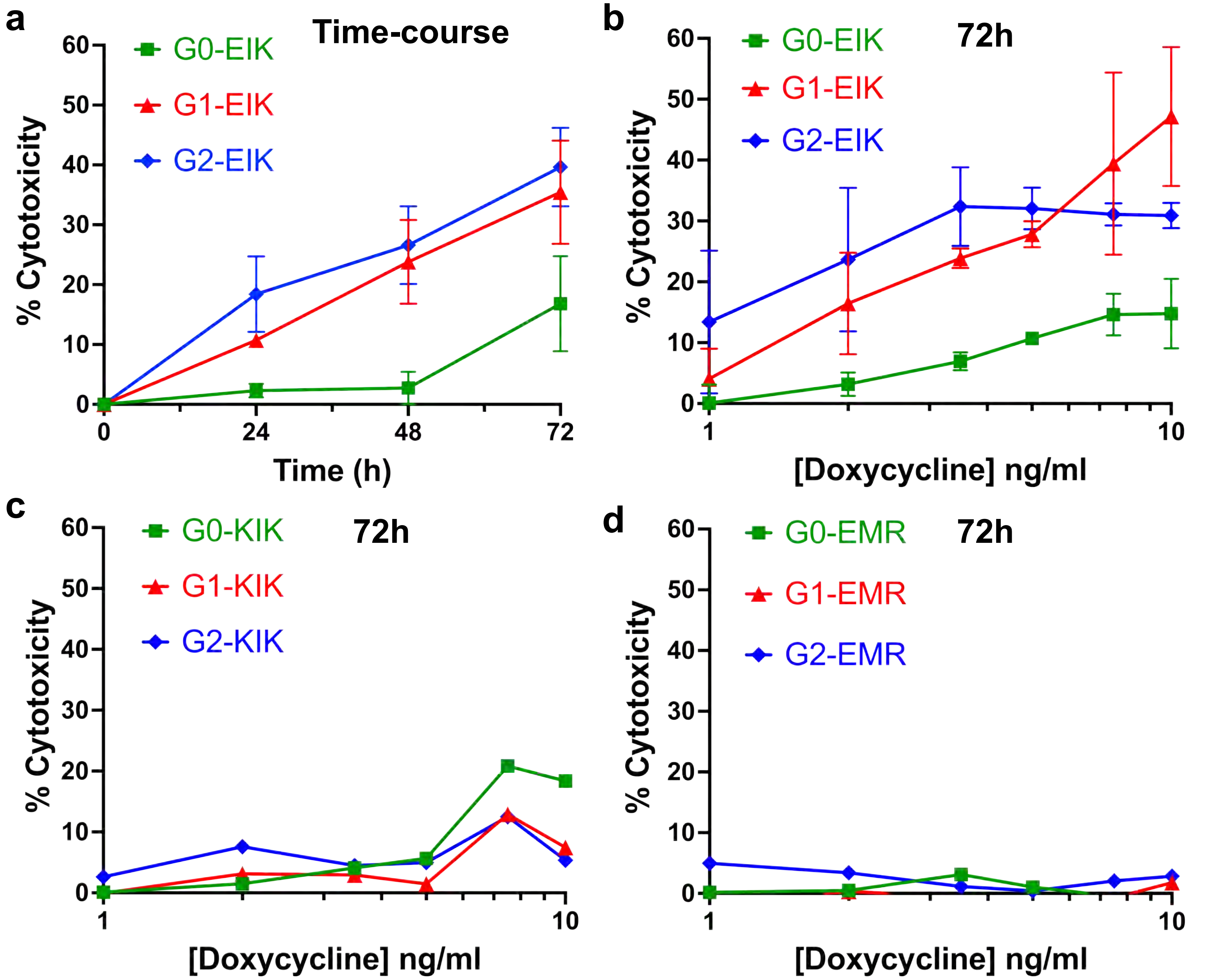

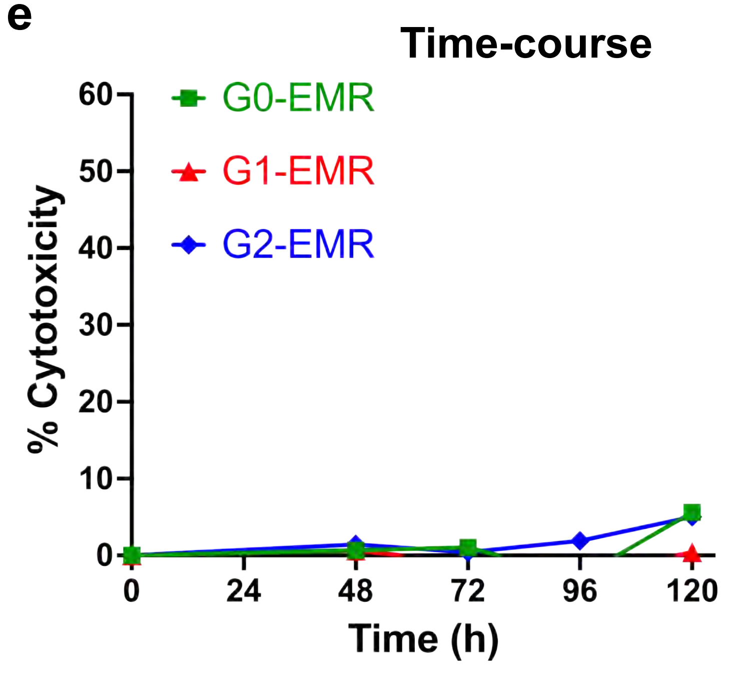

Figure S2. Time-courses and 72h dose-responses of the different haplotypes of iAPOL1 podocytes by CytoTox-Glo^{TM}.

(a) APOL1-G0 EIK exhibits mild latent cytotoxicity. Time-course of EIK podocytes in 5ng/ml dox reveals latent cytotoxicity of G0-EIK starting at 72h, but only at low levels similar to those elicited by G1-EIK and G2-EIK in just 24h.

(b-d) Dox dose responses of iAPOL1-EIK (b), KIK (c) and EMR (d) after 72h exposure, showing mild variant-independent toxicity for KIK clones above 5ng/ml, but none at all for EMR clones.

(e) Time-course of iAPOL1-EMR podocytes induced at 5ng/ml dox confirms complete lack of toxicity for these clones even after 5 days.

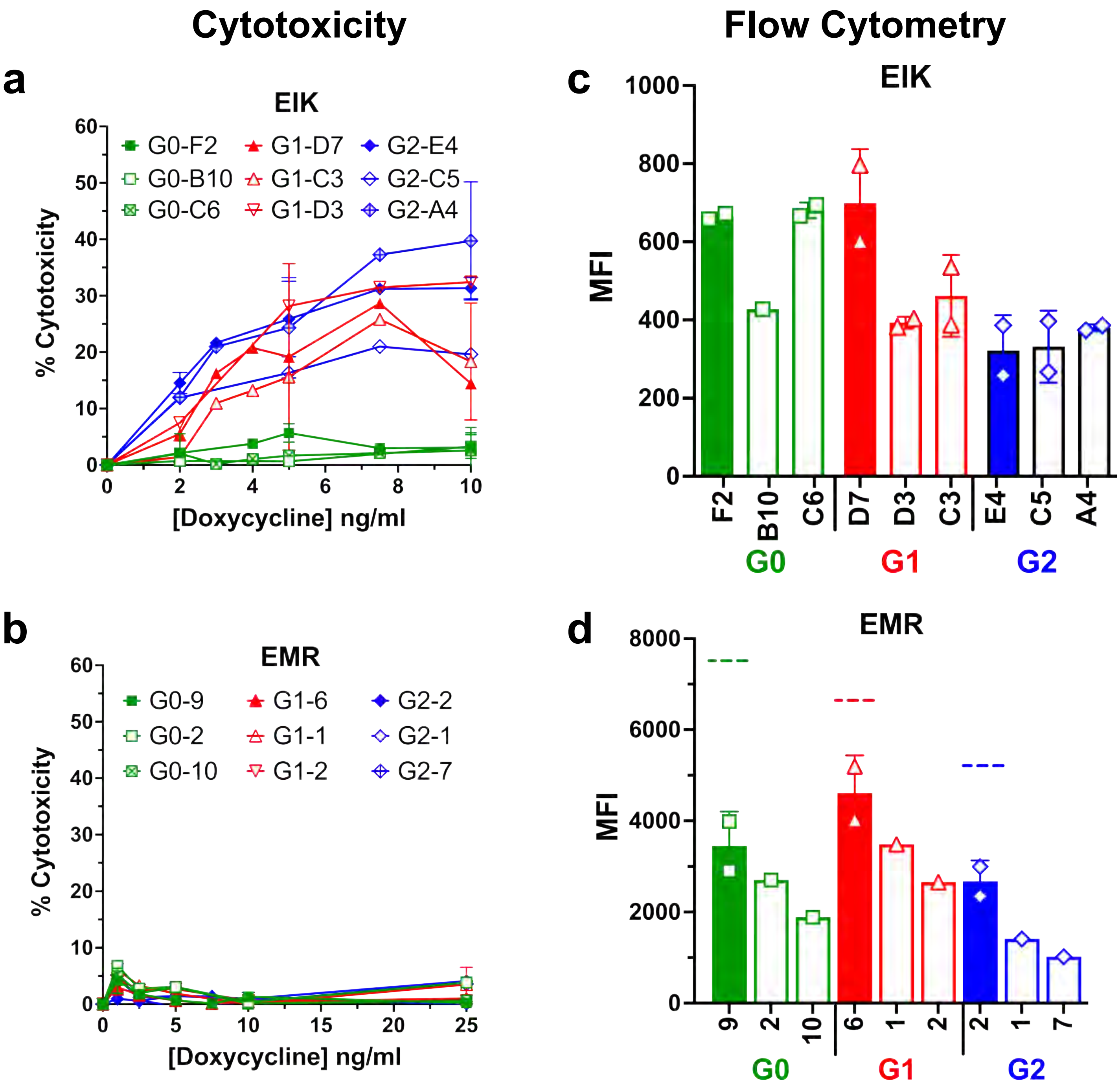

Figure S3. APOL1 cytotoxicity trends are reproduced in independent clones with similar expression, supporting haplotype-specific effects rather than clonal artifacts.

(a,b) CytotoxGlo after 48h induction at 10ng/ml dox, with two different single cell clones (open symbols) in addition to the "lead" clone that was used in all other experiments (solid symbols) for each variant for EIK (a) and EMR (b) haplotypes. Mean and SD of 2 independent experiments is plotted.

(c,d) Flow cytometry of the same (highest expressing of 10 screened) clones as in (a,b) with 2.5μg/ml 3.6D12 then Alexa488 anti-mouse after overnight induction at 10ng/ml dox. The lead clone means are plotted as solid bars, and the others as open bars, with individual experiments overlaid as open symbols.The EMR clones were created after the FACS Calibur flow cytometer was replaced by a FACS Celesta, which gives much higher MFI readings (d). None of the three top EMR clones (bars) attained expression as high as the top EIK clones (dashed lines) analyzed in parallel on the FACS Celesta.

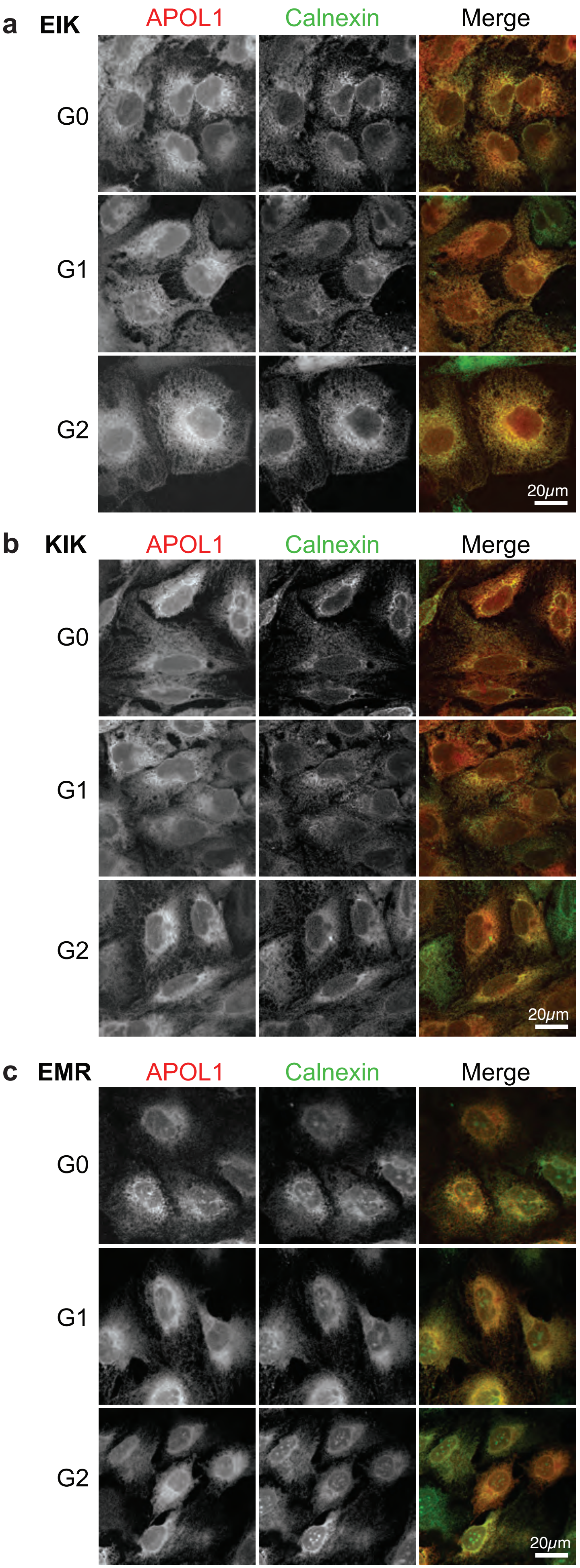

Figure S4. All APOL1 haplotypes localize to the ER. iAPOL1-podocytes expressing G0, G1 and G2 isoform vA in the EIK (a), KIK (b) and EMR (c) haplotype backgrounds after 18h induction with 5ng/ml dox (to minimize cytotoxicity) were triple stained with 4.17A5 anti-APOL1 (red), anti-calnexin cytoplasmic (intracellular) domain (an ER marker, green) and cytochrome C (shown in Figure S5). Data is representative of two or more experiments.

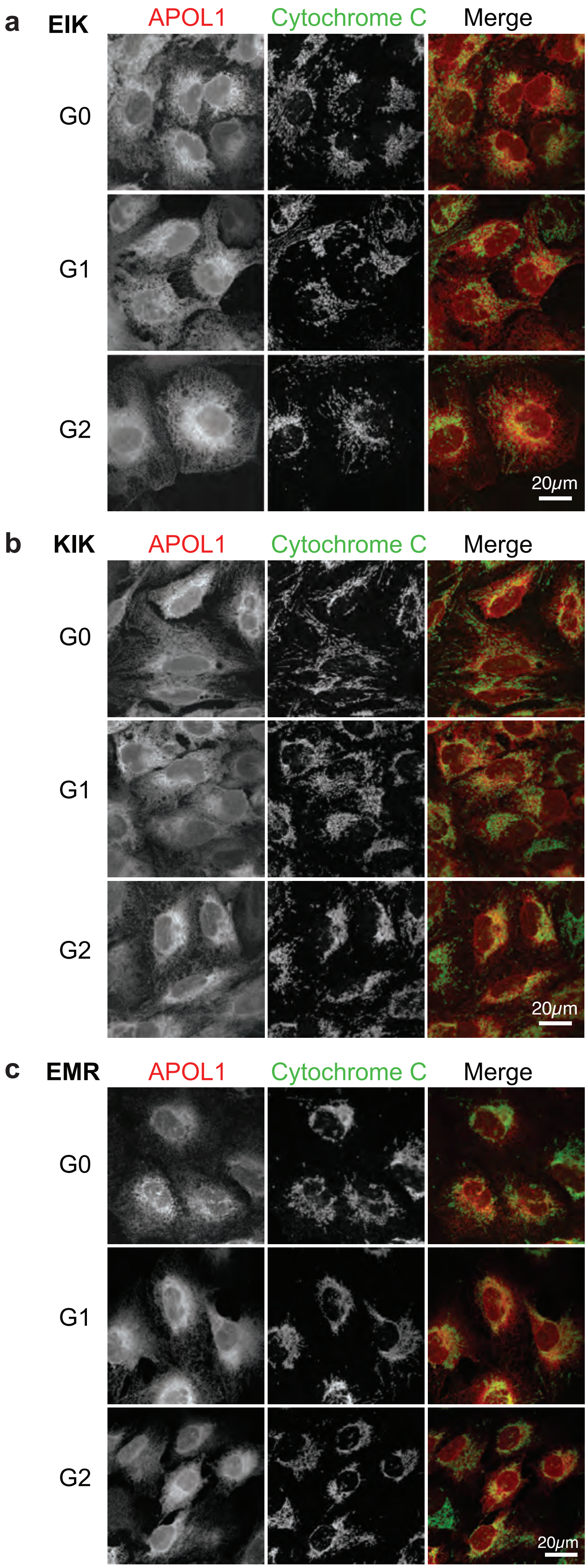

Figure S5. None of the APOL1 haplotypes colocalize with mitochondria.
The exact same iAPOL1 podocytes from Figure S4 are shown overlaid with mitochondrial anti-cytochrome C instead of calnexin the green channel. Data is representative of at least two experiments.

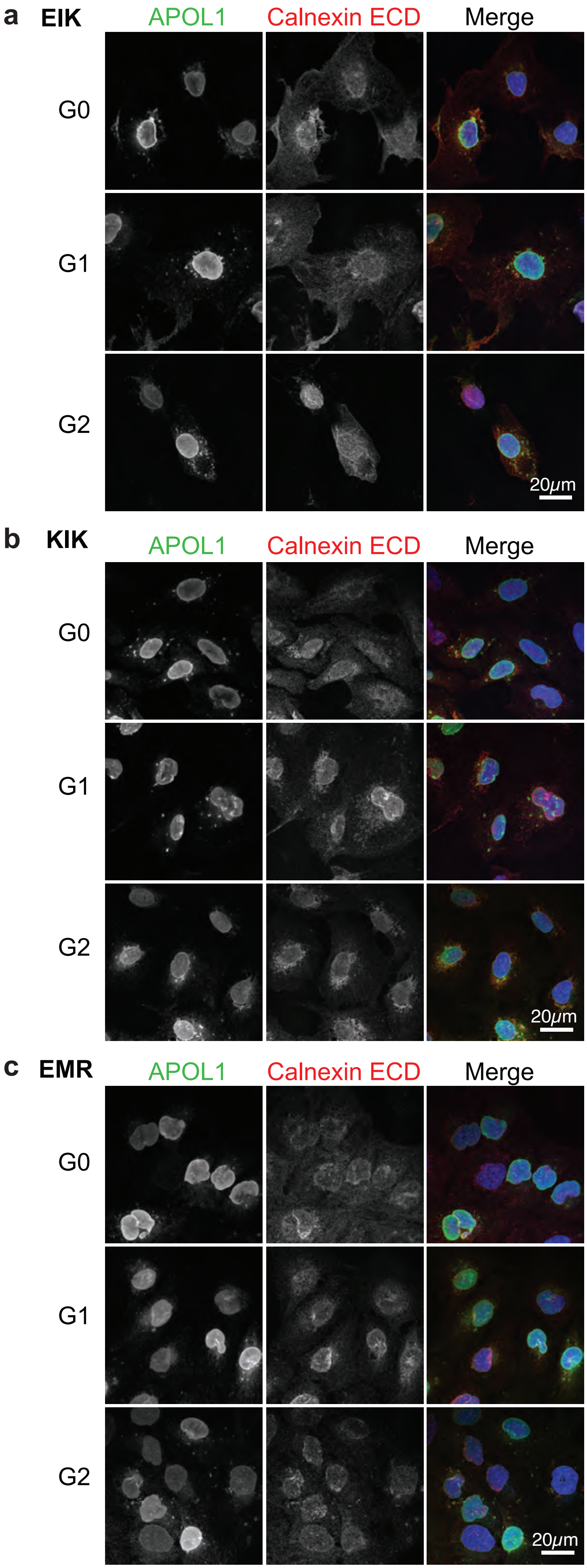

Figure S6. All APOL1 haplotypes have the same topology in the lumen of the ER.
iAPOL1-podocytes expressing G0, G1 and G2 isoform vA in the EIK (a), KIK (b) and EMR (c) backgrounds (after 18h 5ng/ml dox induction) were PFA fixed and the plasma membranes, but not ER membranes, were permeabilized with digitonin prior to co-staining for APOL1 (4.17A5, green) and calnexin luminal domain (red).The nuclear marker DAPI is in the blue channel in the merged images. The APOL1 signal is restricted to the nuclear membrane like the extracellular calnexin epitope instead of the entire reticulum, indicating luminal localization of both (as we have previously published)[19]. The signal is in the nuclear envelope because it is contiguous with the endoplasmic reticulum, but is accessible to antibodies via its nuclear pores (unlike the rest of the endoplasmic reticulum, which was not permeabilized at this concentration of digitonin). Data is representative of two experiments.

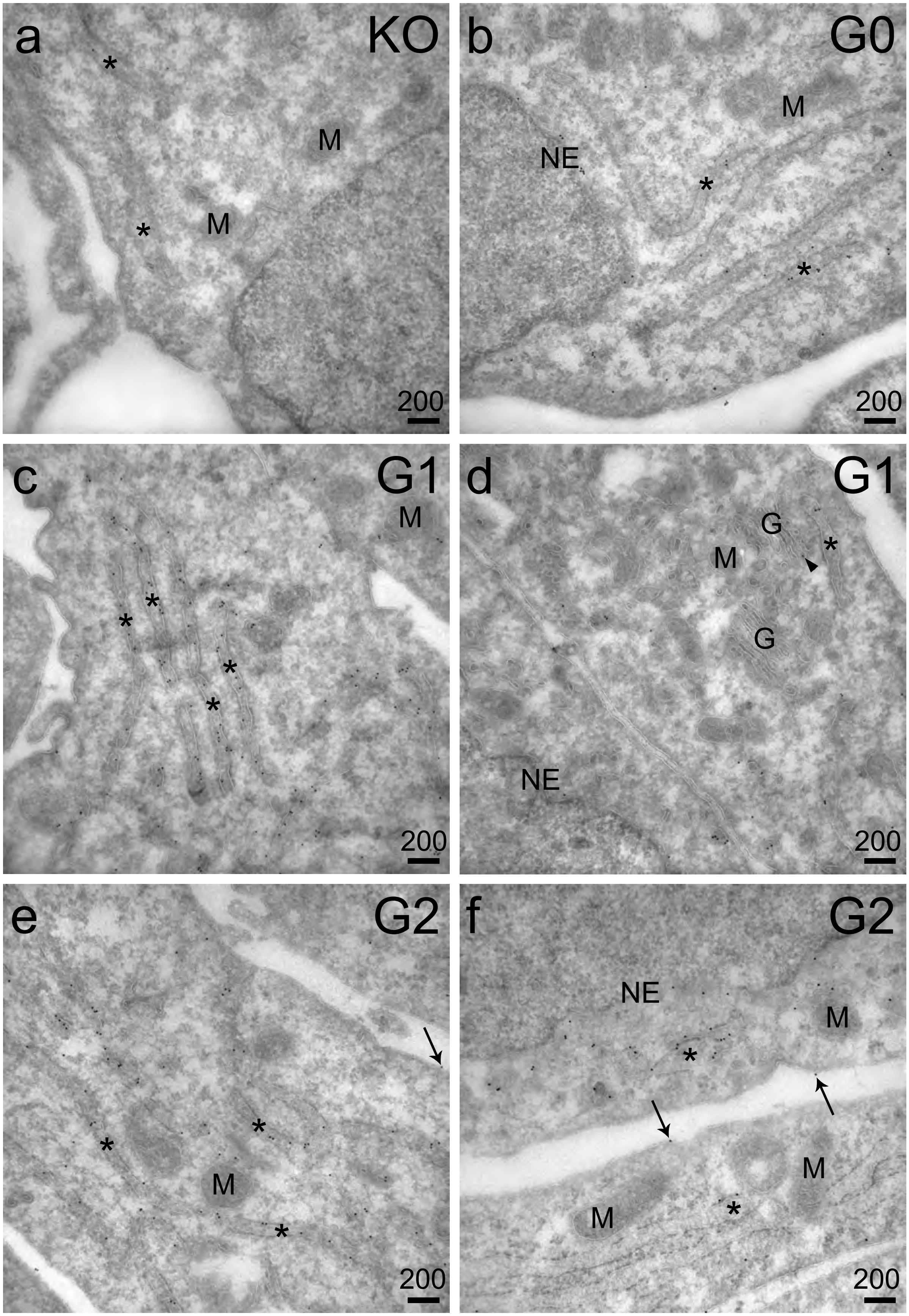

Figure S7. Immunoelectron microscopy confirms no differences between APOL1-G0, G1 and G2-EIK localization. iAPOL1-G0 (b), G1 (c,d) and G2 (e,f) EIK podocytes induced for 8h were immunolabeled with anti-APOL1 3.6E10 followed by protein A-Gold. All three variants are mainly seen in the ER lumen (*), including the nuclear envelope (NE), with lower signals detected in the Golgi (G, arrowhead) and on the plasma membrane (arrows). Mitochondria (M) were devoid of signal, as expected from the immunofluorescence (Fig. S5) and previous results with G0-KIK[19]. Specificity of 3.6E10 was confirmed by the lack of signal seen in uninduced G2 podocytes (a, KO). Scale bars are 200nm (5000x).

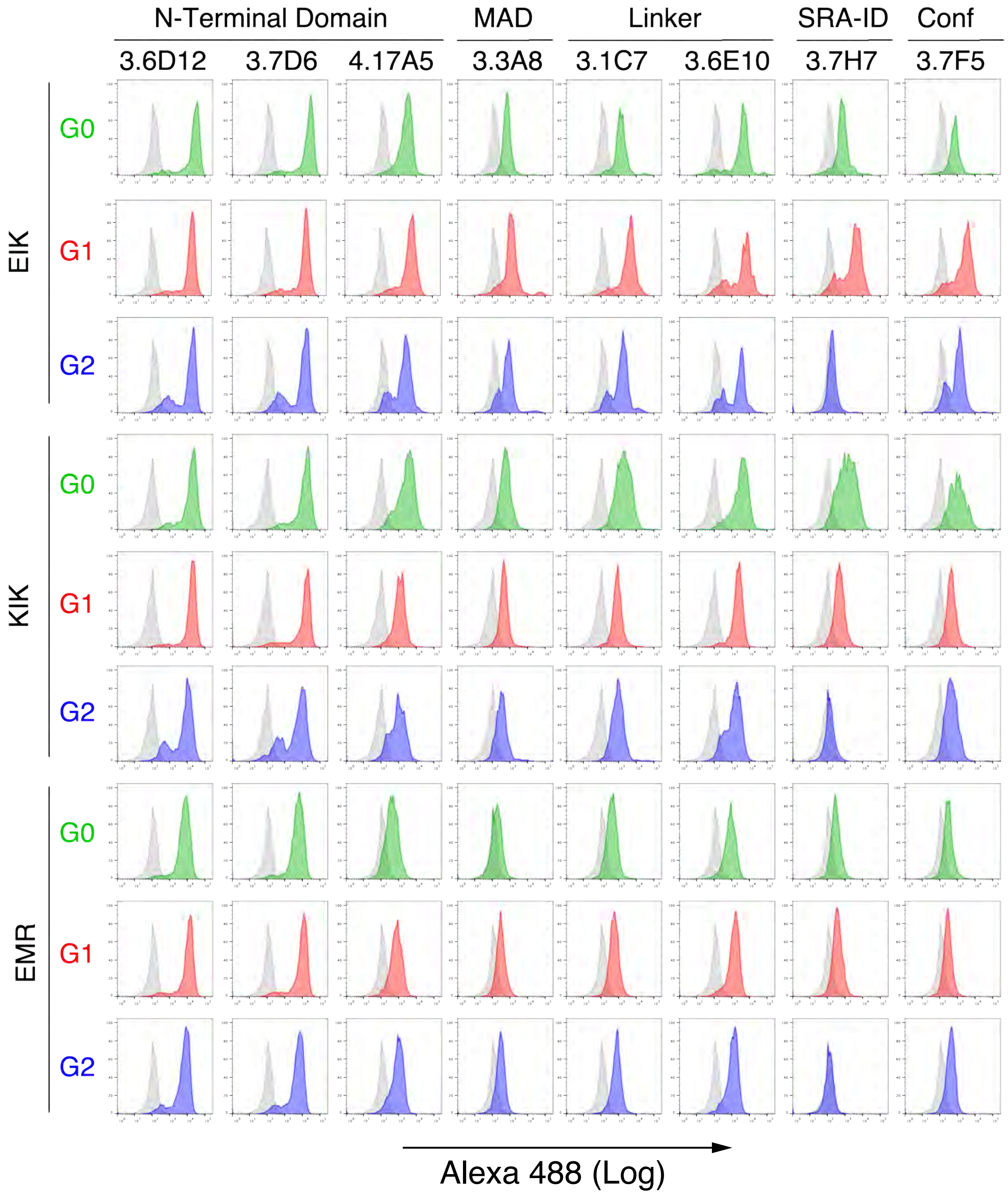

Figure S8. All haplotypes exhibit similar surface topology by flow cytometry.
Flow cytometry of induced iAPOL1-podocytes with a panel of mouse monoclonals to different domains of APOL1. Grey histograms are Alexa488-anti-mouse alone and colors are the indicated anti-APOL1 signals (G0, G1 and G2). Of particular interest, the linker domain antibodies 3.1C7 and 3.6E10 show no difference between haplotypes. MAD, membrane addressing domain; SRA-ID, SRA-interacting domain (note 3.7H7 to this domain cannot detect G2); Conf, conformational (only detects full-length APOL1). Data is representative of two or more experiments.

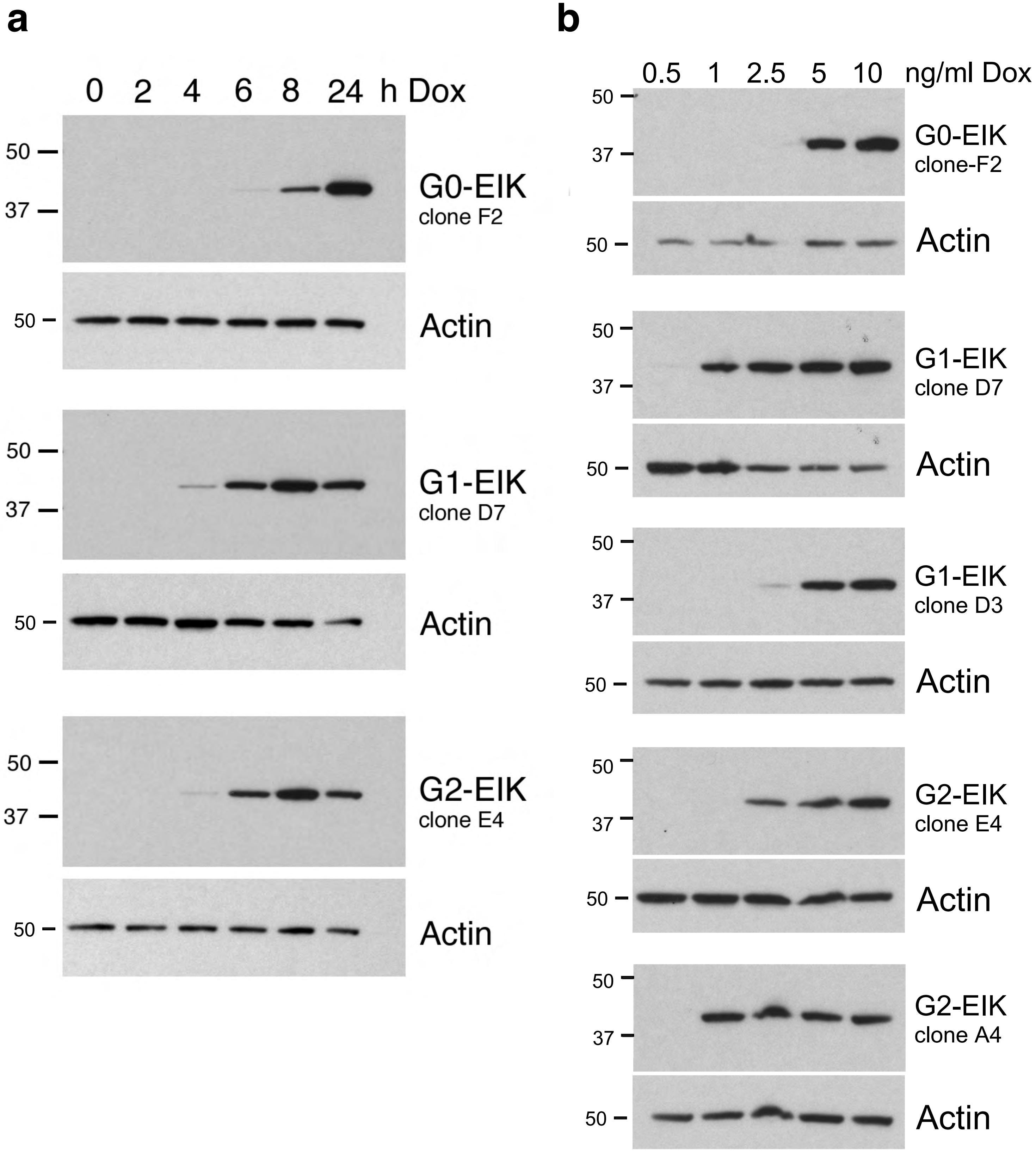

Figure S9. APOL1-G1 and G2-EIK express more readily than G0.
(a) Western blot of of 10μg lysates of APOL1 in iAPOL1-EIK podocytes at different times after induction with 5ng/ml dox showing high-expressing “lead” clones G1 (clone D7) and G2 (clone E4) express APOL1 2h earlier than G0 (clone F2). Actin is the loading control. Full western blots (each on a different gel for each clone) are shown in Supplementary Fig. 24.
(b) Western blot of iAPOL1-EIK podocytes (10μg lysates) induced for 24h at the indicated dox concentrations, showing the lead and backup clones of G1 and G2 both start expressing detectably at 1-2.5ng/ml dox, compared to 5ng/ml for G0. These expression trends are similar to those detected by the more sensitive FACS technique (Figure S1). Loading control is actin. The two G1 clones were run on one gel, the two G2 clones on another, and G0-F2 on a different blot (see full blots in Supplementary Fig. 25). Anti-APOL1 was a 0.05μg/ml mixture of rabbit monoclonals 3.1C1 and 3.7D6 (ref. 19).

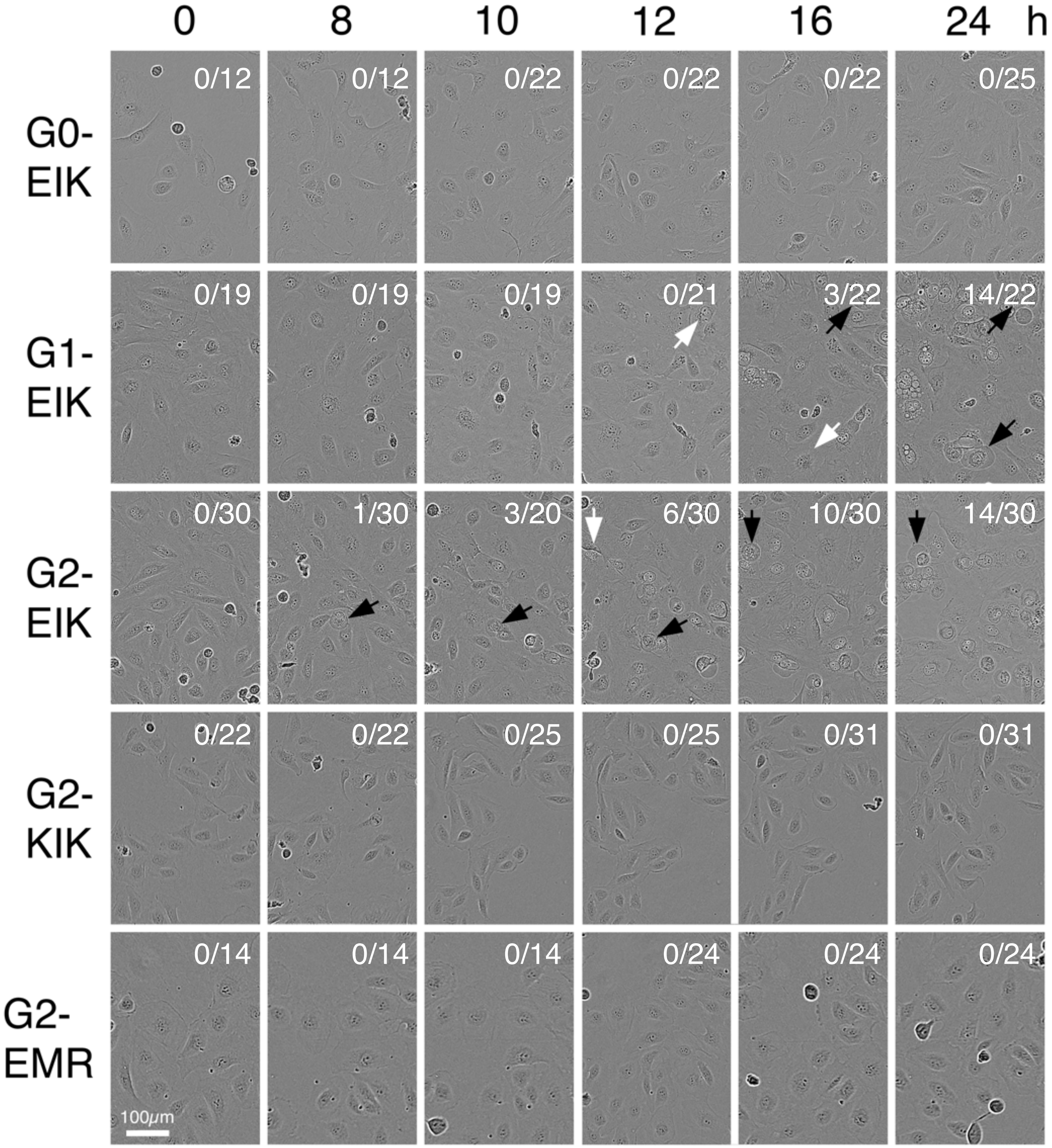

Figure S10. Podocyte cell swelling occurs at least 2h after arrival of APOL1-EIK at the cell surface. Phase contrast microscopy (using IncuCyte®) of iAPOL1 podocytes at the indicated hours after induction by 5ng/ml dox. G1-EIK podocytes start swelling after 16h, G2-EIK podocytes after 8h and the others not at all within 24h. Arrows pointing in the same direction indicate the same cell over time; black arrows indicate cell swelling, which is preceded by the nuclear membrane becoming more prominent (white arrows). White numbers indicate the number of swollen cells over the number of cells shown in each panel. Data are representative of 4-6 experiments.

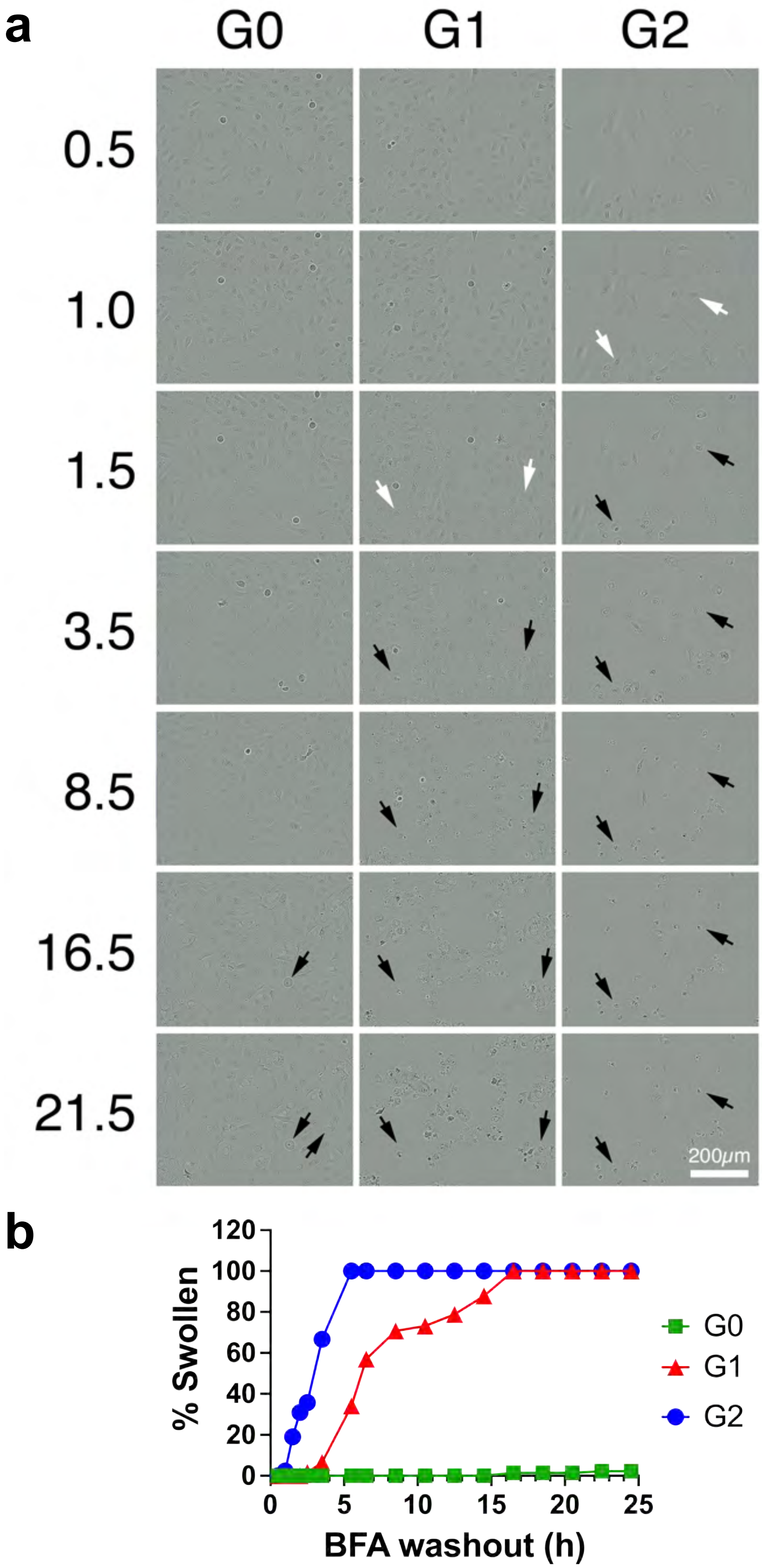

Figure S11. iAPOL1-EIK podocyte swelling timecourse after BFA washout.
(a) iAPOL1-EIK podocytes were treated for 24h with 25ng/ml Dox and 5μg/ml BFA, then both drugs were washed out and the cells were imaged by phase contrast every 30 mins. Cropped stills of timepoints (h) selected to illustrate the milestones in swelling for each variant (1.5h onset for G2 with most cells swollen by 3.5h and all by 5.5h; 3.5h onset for G1 with most swollen after 8.5h and all by 16.5h; and 16.5h onset for G0 with still only a few cells swollen by 21.5h). Black arrows pointing in the same direction follow selected swollen cells over time, whereas the white arrows show the same cells prior to the onset of swelling.
(b) Quantitation of % swollen cells every 1-2h post BFA washout for the entire (uncropped) field of cells in this experiment.

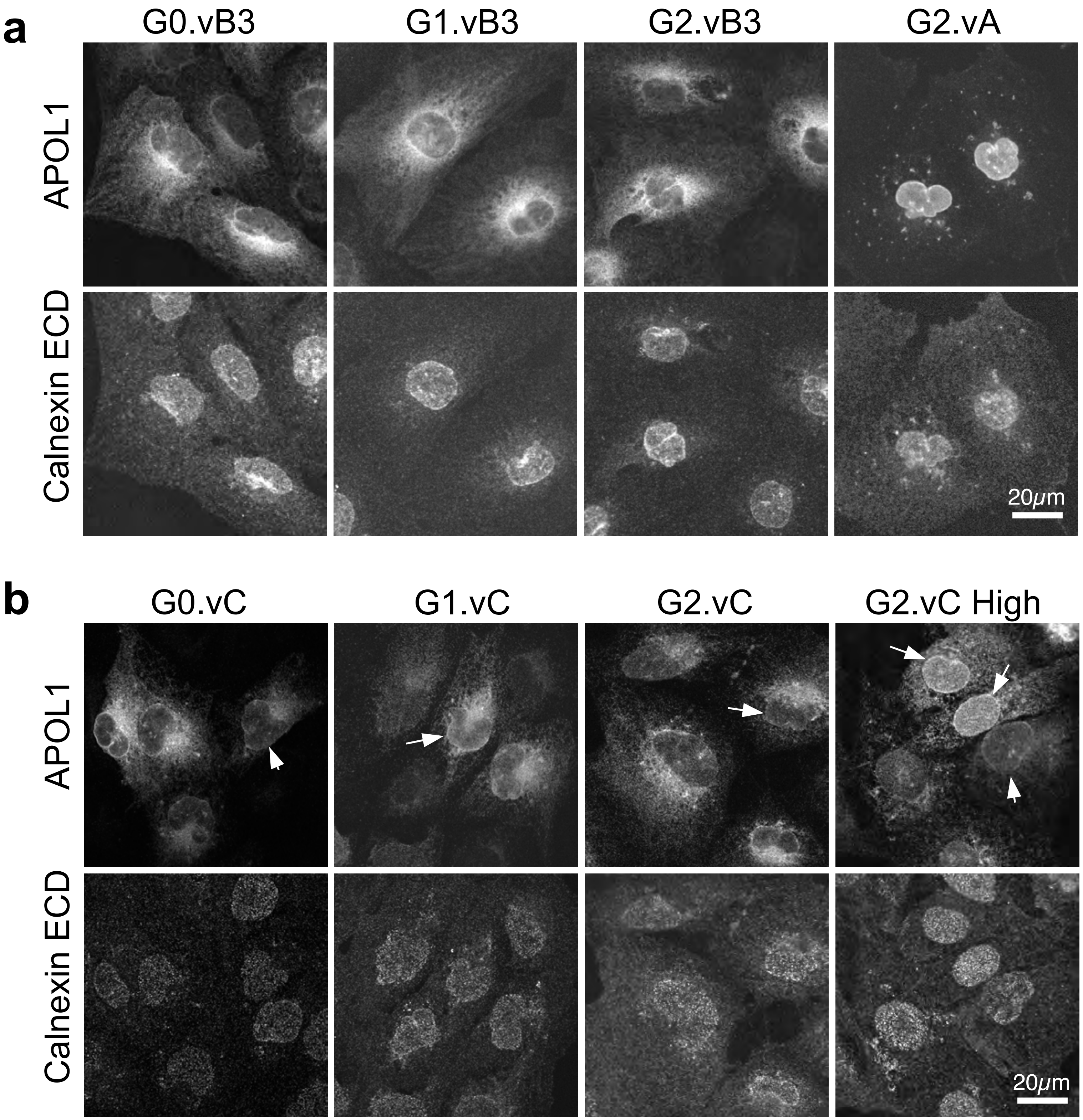

Figure S12. Topology of APOL1-G1 and G2 isoforms vB3 and vC.
iAPOL1-podocytes expressing G0, G1 and G2 (EIK) isoforms vB3 (a), and vC (b) were stained for APOL1 (top rows, 2μg/ml 4.17A5) using the PFA/digitonin protocol, which does not permeabilize the ER membrane, which was confirmed by nuclear instead of reticular staining for the ER-luminal calnexin extracellular domain (ECD; bottom rows, clone 37 at 2.5μg/ml (a) or 1.25μg/ml (b)). All three variants of APOL1.vB3 give cytoplasmic staining, in contrast to the luminal nuclear membrane staining of APOL1-G2.vA comparator (top right). All three variants of vC are mostly cytoplasmic, but a portion is also luminal (arrows). The luminal signal is more pronounced in the G2.vC high clone that expresses more highly and causes low levels of cytotoxicity (see Figure 6a,c).

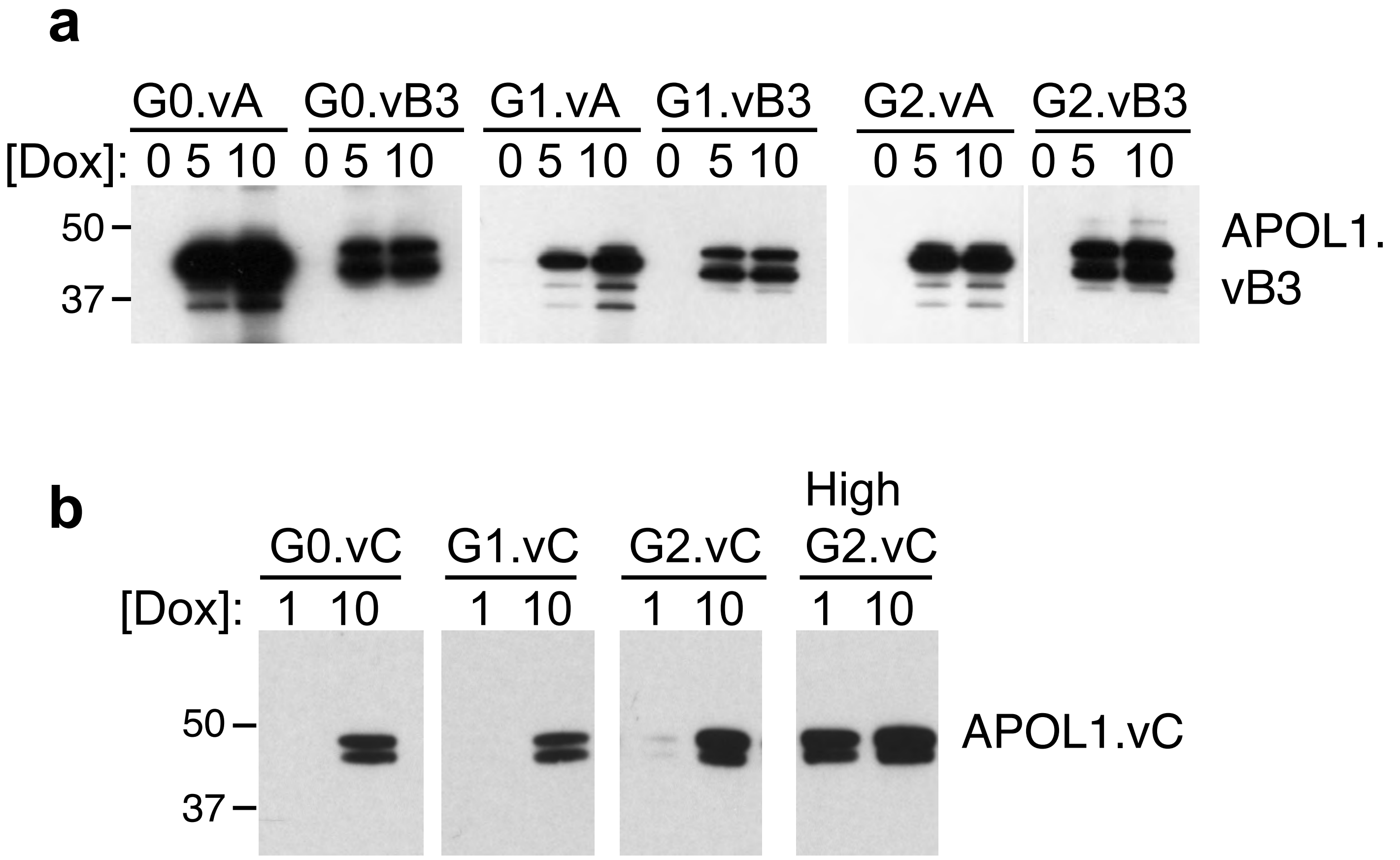

Figure S13. APOL1 Western blots of different isoforms showing total expression levels.
(a) Western blotting of the APOL1.vB3-EIK isoforms induced for 16h at the indicated dox concentrations (in ng/ml), showing similar or higher total expression than the corresponding vA-EIK isoform. The doublet bands likely indicate cytoplasmic cleavage, as previously noted [14,19,33]. G0, G1 and G2.vB3 were run on separate blots with corresponding isoform vA for comparison. The white gap before G2.vB3 indicates removal of intervening lanes on the same blot of another clone. See Supplementary Fig. 26 for original scans.
(b) Western blotting of APOL1.vC-EIK isoforms induced for 16h with 1 or 10ng/ml dox. The doublet likely represents incomplete processing of the signal sequence. The two G2 clones were run on the same blot, but G0 and G1 were run on different blots - see raw scans in Supplementary Fig. 27. A mixture of 0.05μg/ml anti-APOL1 rabbit monoclonals 3.7D6 and 3.1C1 (ref. 19) was used for both blots.

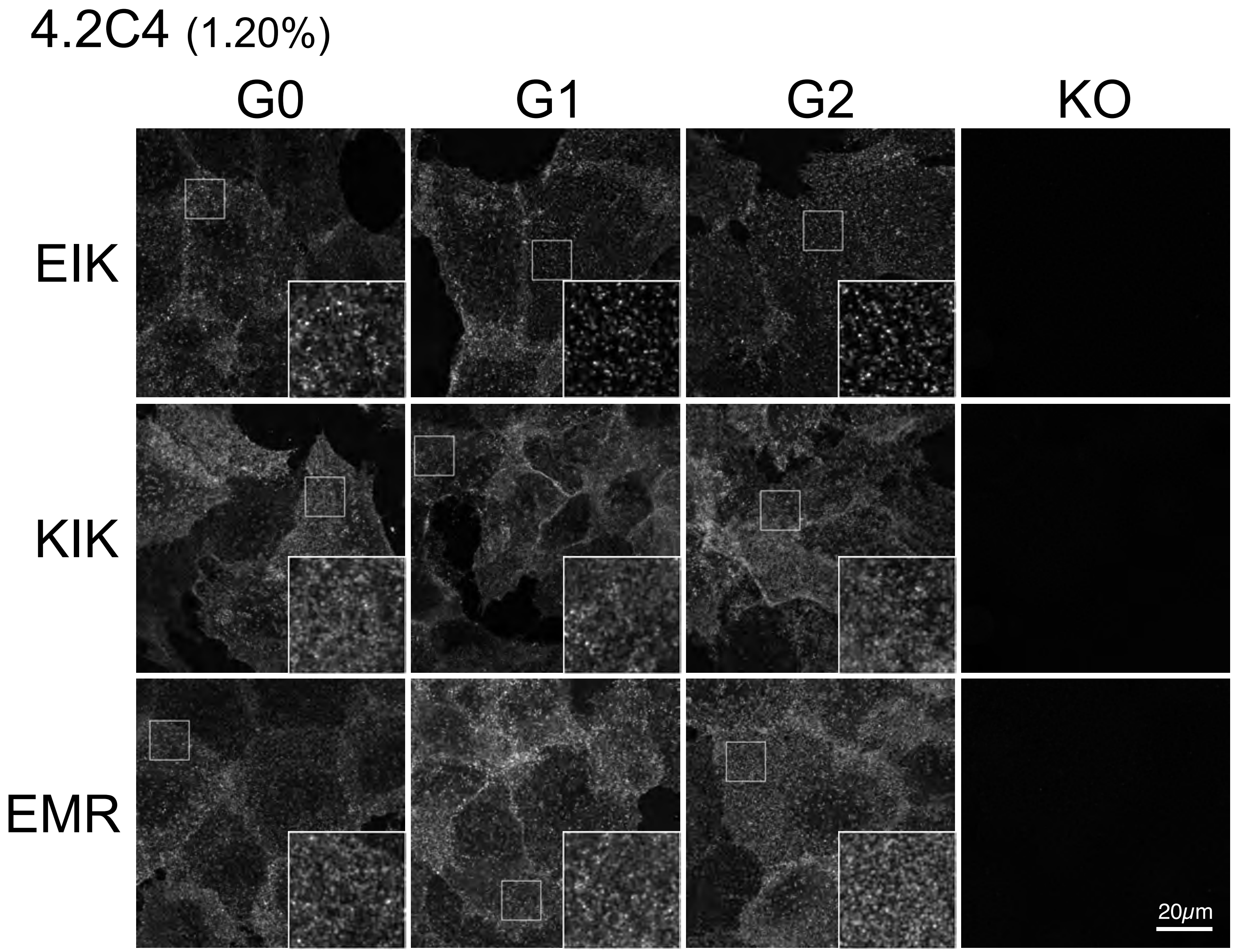

Figure S14. APOL1-EIK clusters more completely on the podocyte surface than KIK or EMR with a different APOL1 antibody.

As in Figure 7, but with an antibody (4.2C4) to a different N-terminal domain epitope than 3.6D12, also with minor (1.20%) aggregation. iAPOL1-podocytes (KO are uninduced G2) were incubated for 1h on ice with 5μg/ml 4.2C4, then warmed for 15 min to induce any internalization. Cells were then washed, fixed, Triton-X-100 permeabilized and stained with Cy3-anti-mouse. Insets are 3x magnification of the boxed regions. As seen with 3.6D12, 4.2C4 more completely clusters G1 and G2-EIK than the other haplotypes, with G0-EIK exhibiting clustering intermediate between G1/G2-EIK and the non-toxic haplotypes.

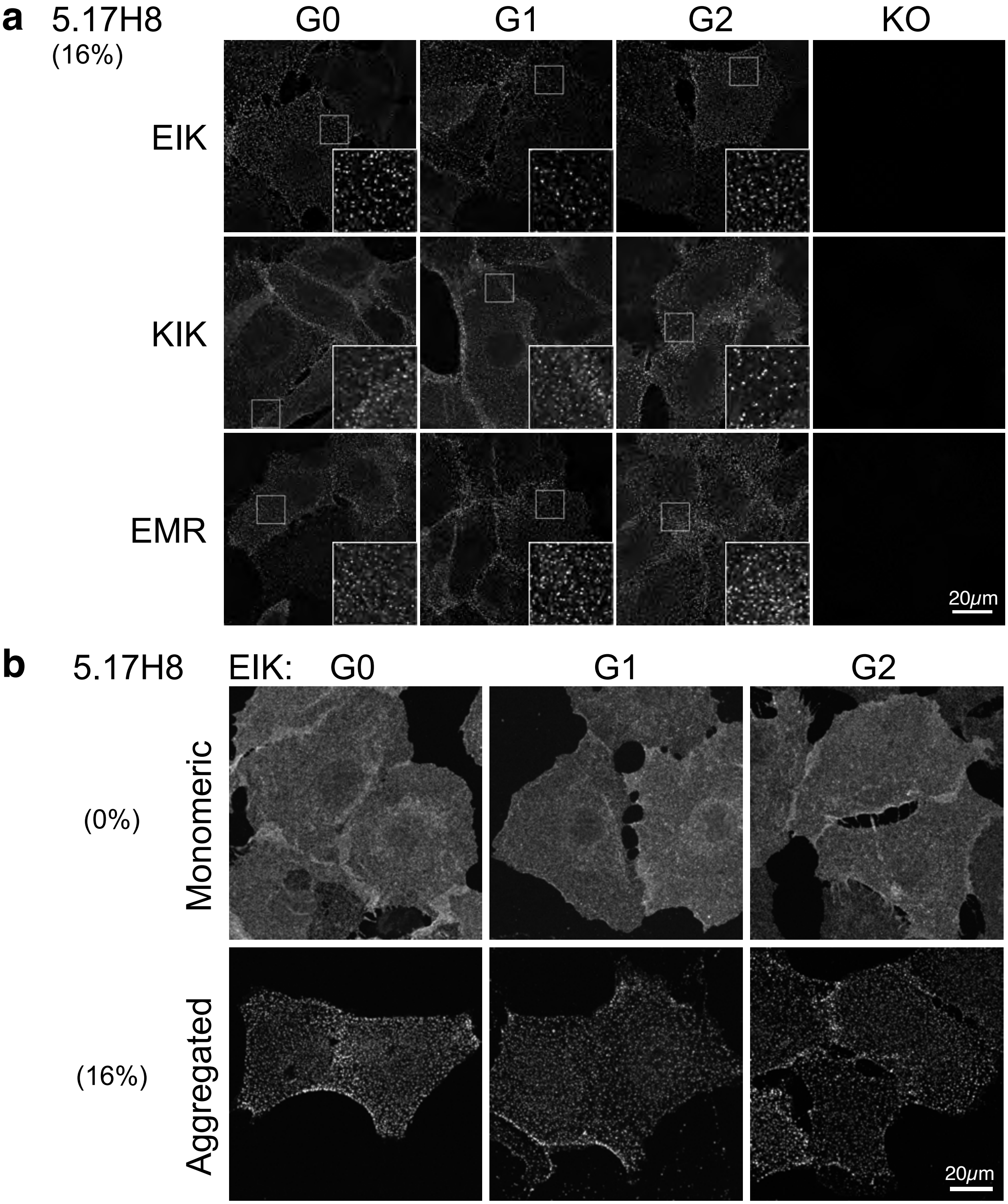

Figure S15. APOL1 surface clusters are more pronounced with highly aggregated anti-APOL1 monoclonal 5.17H8.
We took advantage of a screening prep of rabbit monoclonal 5.17H8, which was only single column purified and had 16% aggregate, thus failing our usual quality control limit of 5%. Aggregated 5.17H8 was incubated at 5μg/ml with iAPOL1-podocytes on ice for 1h (without warming), washed, fixed and detected with Alexa488 anti-rabbit following Triton-X-100 permeabilization. Surface clustering is more pronounced for all haplotypes than with the minimally aggregated 3.6D12 and 4.2C4 monoclonals in Fig. 7 and Supplementary Fig. 14. With 5.17H8, APOL1-EMR exhibited clustering intermediate between EIK and KIK, suggesting residue E150 may be important for clustering, despite the 5.17H8 epitope covering aa 63-78. Insets show 3x magnification of the main boxed areas. Experiment was repeated three times.
b) Non-aggregated 5.17H8 does not cluster APOL1. iAPOL1-EIK podocytes were incubated with 5μg/ml 0% aggregated (100% monomeric; top row) or 16% aggregated (bottom row) 5.17H8 anti-APOL1 for 1h on ice, followed by 5 min at 37°C. Non-aggregated 5.17H8 does not cluster APOL1, confirming that clustering depends on antibody aggregation rather than epitope. Representative of two experiments.

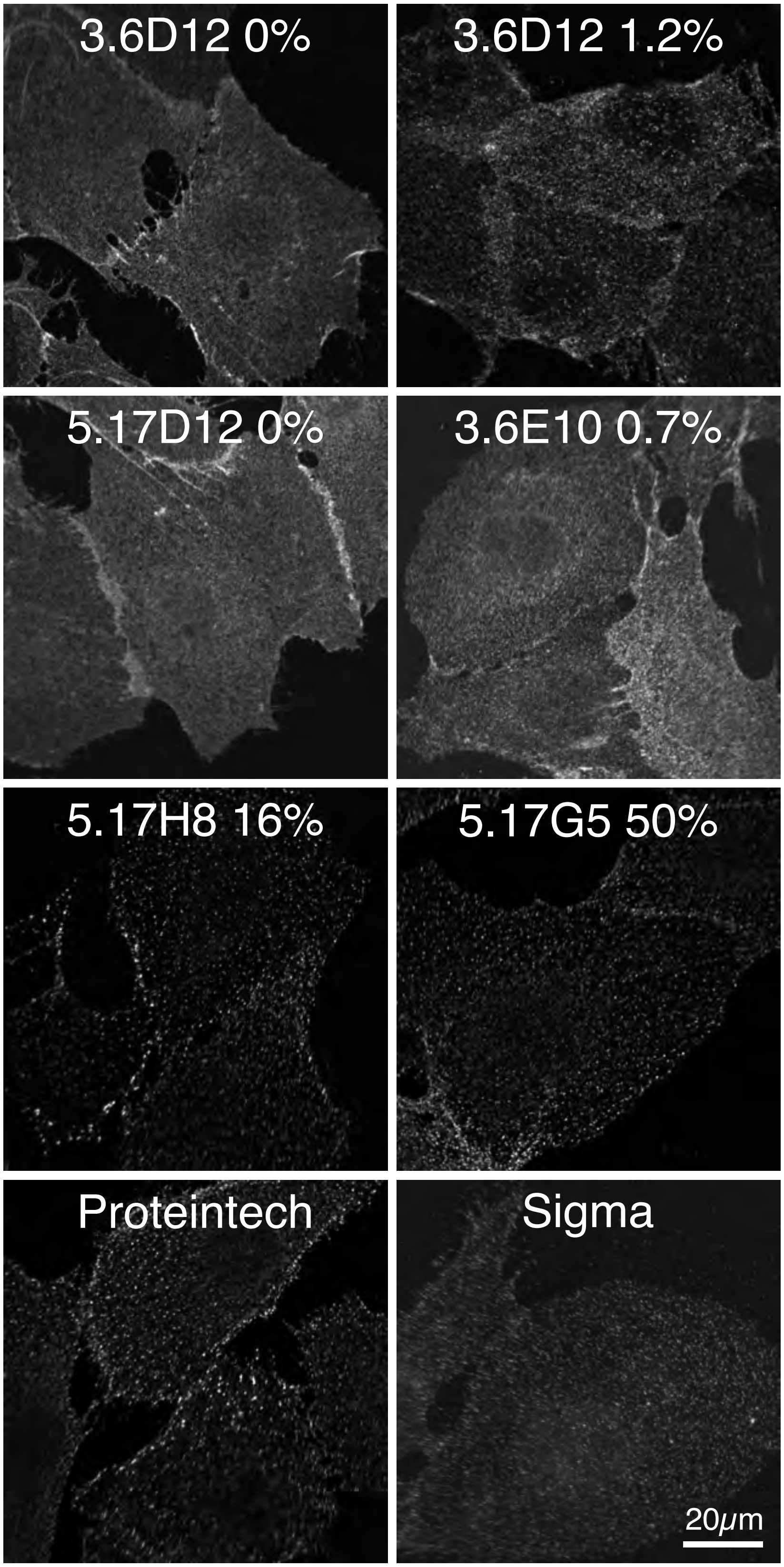

Figure S16. APOL1 surface clustering correlates with aggregation, not epitope. iAPOL1-G2-EIK podocytes were incubated for 1h on ice with 5μg/ml of the indicated antibodies (% aggregation listed) without warming, then fixed, saponin permeabilized and stained with secondary antibodies. Antibodies are against distinct (3.6D12, 5.17D12) or overlapping (5.17H8, 5.17G5) epitopes in the N-terminal domain (including the Proteintech polyclonal raised to aa 28-238), while 3.6E10 recognizes the linker and the Sigma polyclonal was raised to the MAD and SRA-ID (aa 263-387). APOL1-G2 EIK clustering occurs with as little as 0.7% aggregate and is much more pronounced at ≥16%. The aggregation status of the commercial polyclonals is unknown.

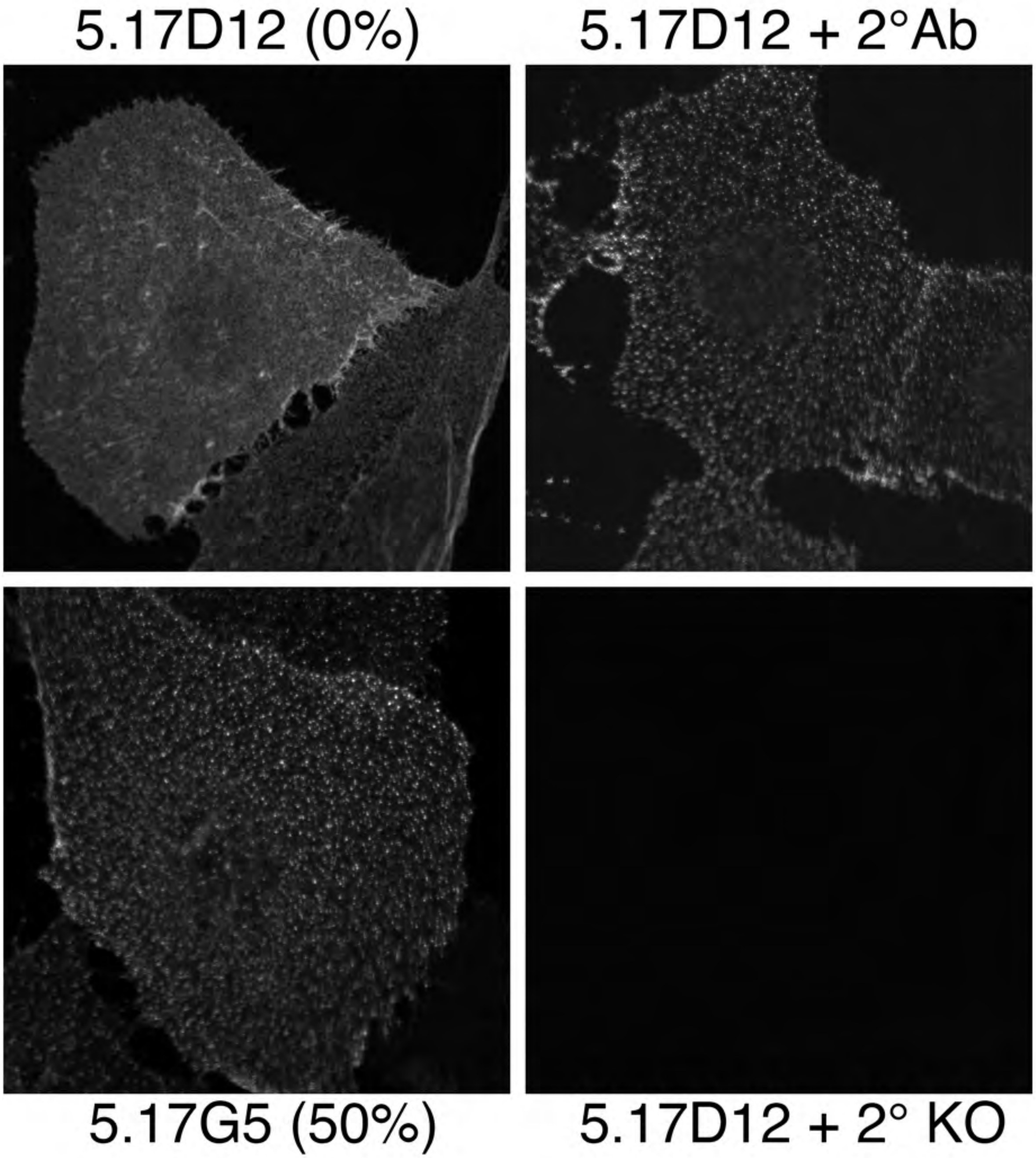

Figure S17. Secondary antibody-mediated clustering of monomeric anti-APOL1. iAPOL1-G2 EIK podocytes were incubated with 5µg/ml 0% aggregated 5.17D12 for 30 min on ice, then washed and cross-linked (or not) with 1µg/ml Alexa488 anti-rabbit for 30 min on ice. Monomeric 5.17D12 exhibited smooth staining, whereas when cross-linked with the secondary antibody, it formed large clusters similar to 5.17G5 (50% aggregated) without the secondary antibody. Secondary antibody-mediated APOL1 clustering was specific because there was no signal on uninduced G2-EIK podocytes (KO). Data are representative of 2 experiments.

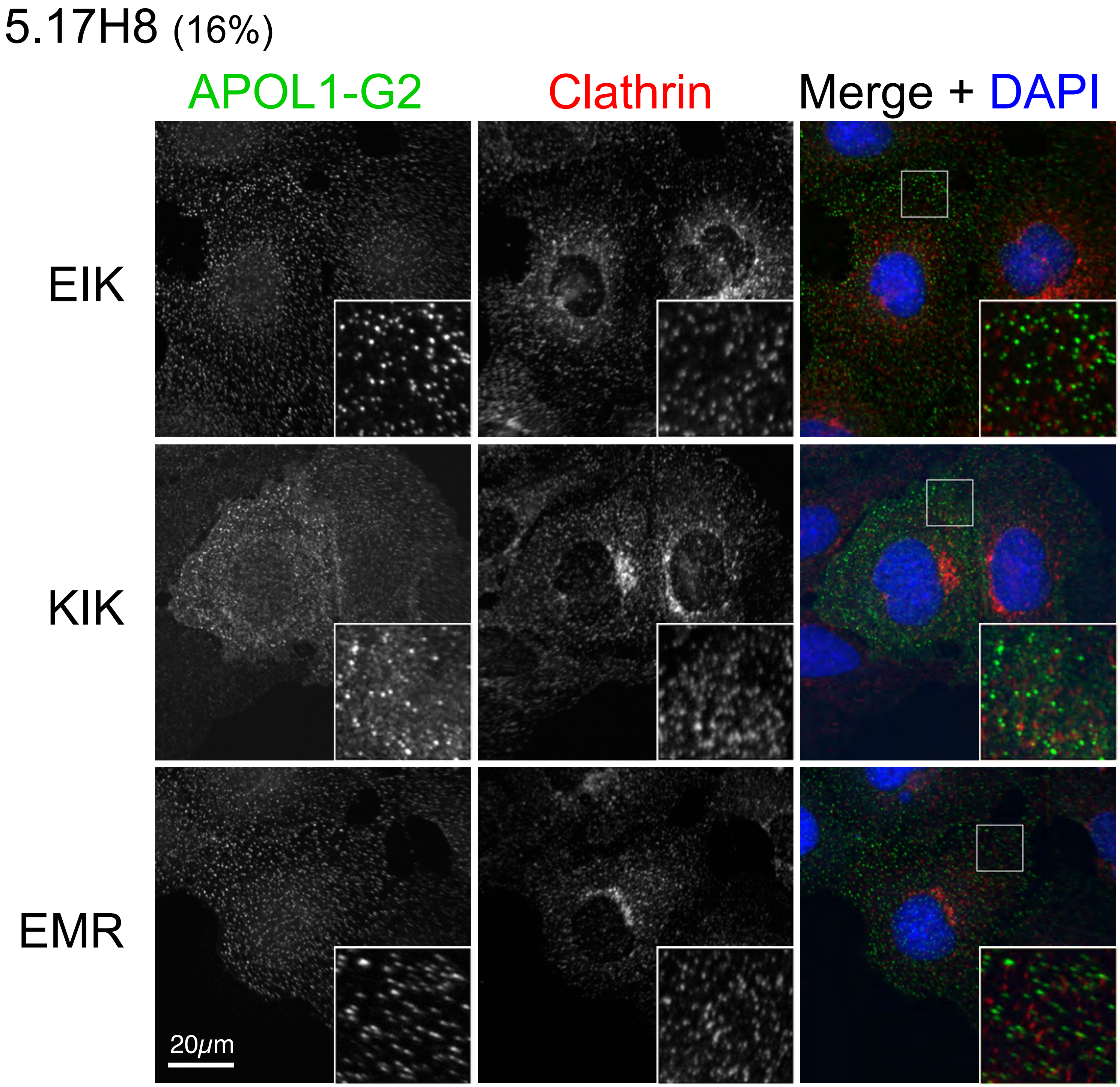

Figure S18. APOL1-G2 large (5.17H8-mediated) clusters are not in clathrin-coated pits. iAPOL1-G2 EIK, KIK and EMR podocytes incubated with 16% aggregated 5.17H8 (green) for 1h on ice, then warmed for 5 min to trigger coated vesicle formation, were stained (post fixation and saponin permeabilization) for clathrin with monoclonal X22 (red). There was no significant colocalization between APOL1 and clathrin, thus APOL1-G2 puncta are not in clathrin-coated pits or vesicles. Insets are 3x magnification of the boxed areas. Data are representative of 2-3 experiments.

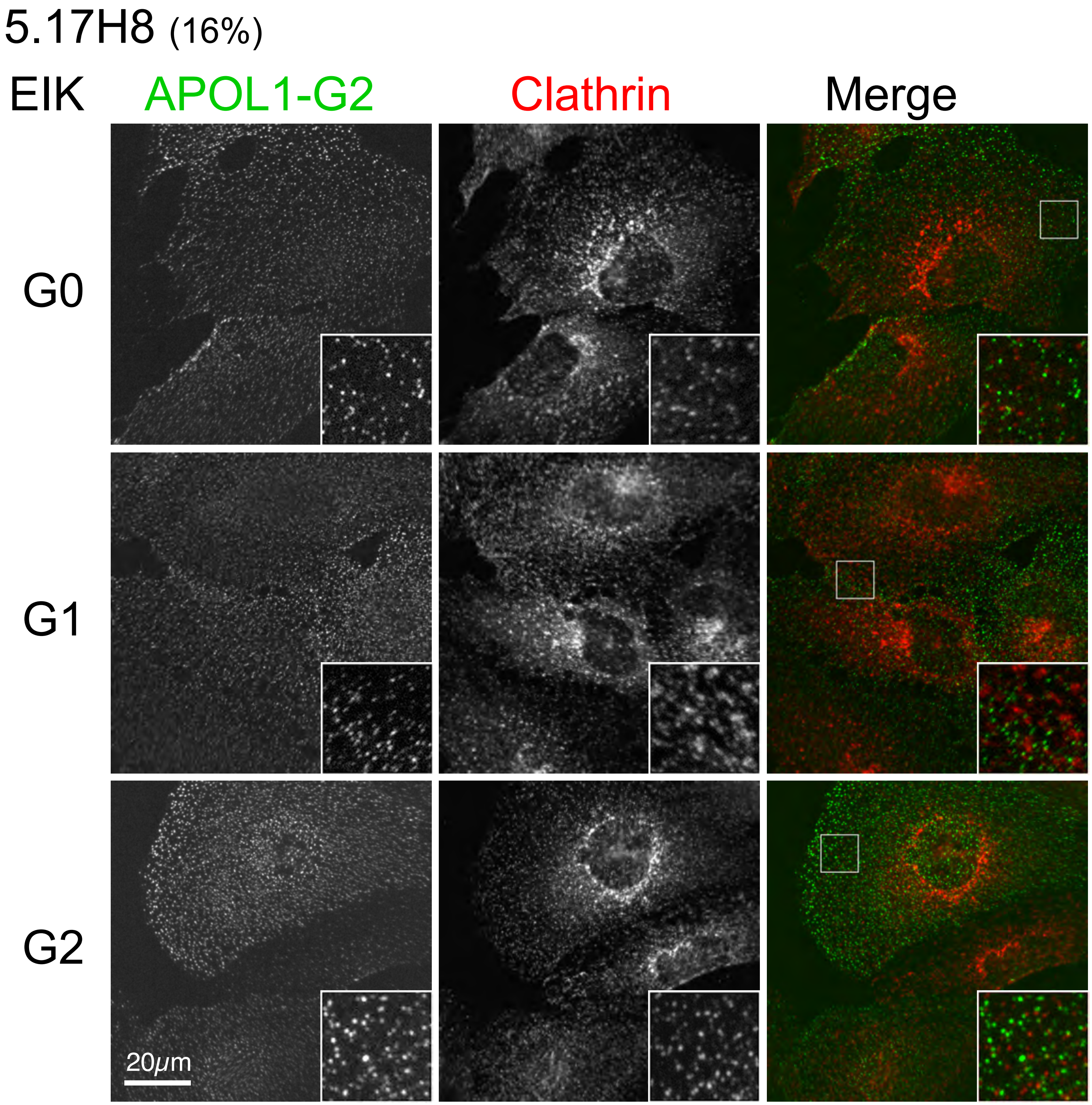

Figure S19. APOL1-EIK large (5.17H8) clusters are not in clathrin-coated pits.
To ensure the large 5.17H8-mediated clusters were not unique to APOL1-G2, iAPOL1-G0, G1 and G2-EIK podocytes were incubated for 1h on ice then warmed for 5 min with 16% aggregated 5.17H8 (green), then stained for clathrin with monoclonal X22 (red). All the APOL1-EIK variants clustered strongly, but none overlapped with clathrin, thus the APOL1 puncta do not represent clathrin-mediated endocytosis. Insets are 3x magnification of the boxed areas.

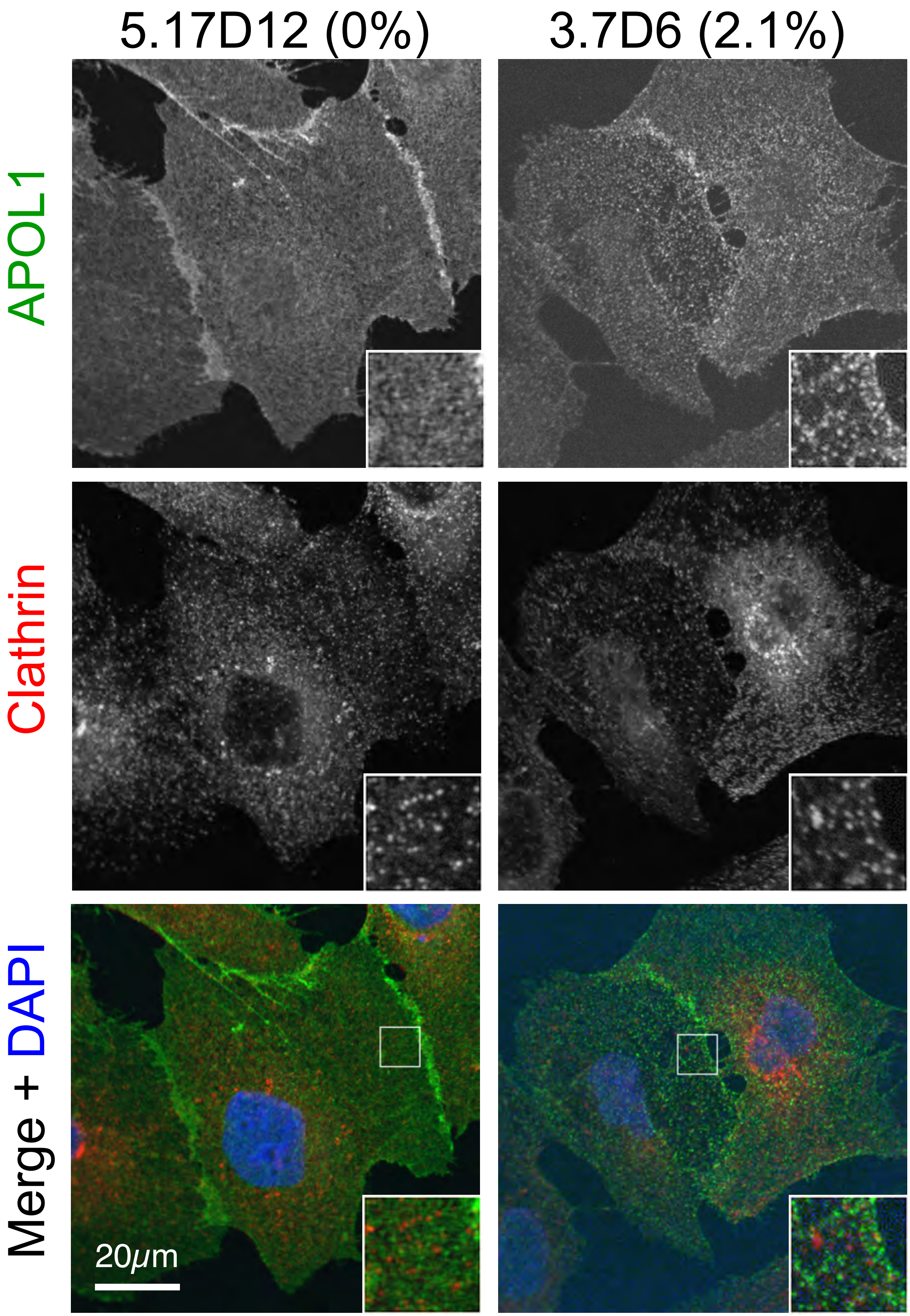

Figure S20. Smooth APOL1 and small APOL1 clusters are not in clathrin-coated pits. iAPOL1-G2 EIK podocytes incubated for 1h on ice (with no warming afterwards) with 0% aggregated rabbit 5.17D12 (left) or 2.1% aggregated mouse 3.7D6 (right; green). After washing, fixing, and saponin permeabilizing, cells were stained for clathrin with monoclonal X22 (left) or rabbit anti-clathrin (right; red). Insets are 3x magnification of the boxed areas. Neither the smooth monomeric APOL1 (5.17D12) nor the small 3.7D6 APOL1-mediated clusters colocalize with clathrin. Data were reproduced in one or more experiments.

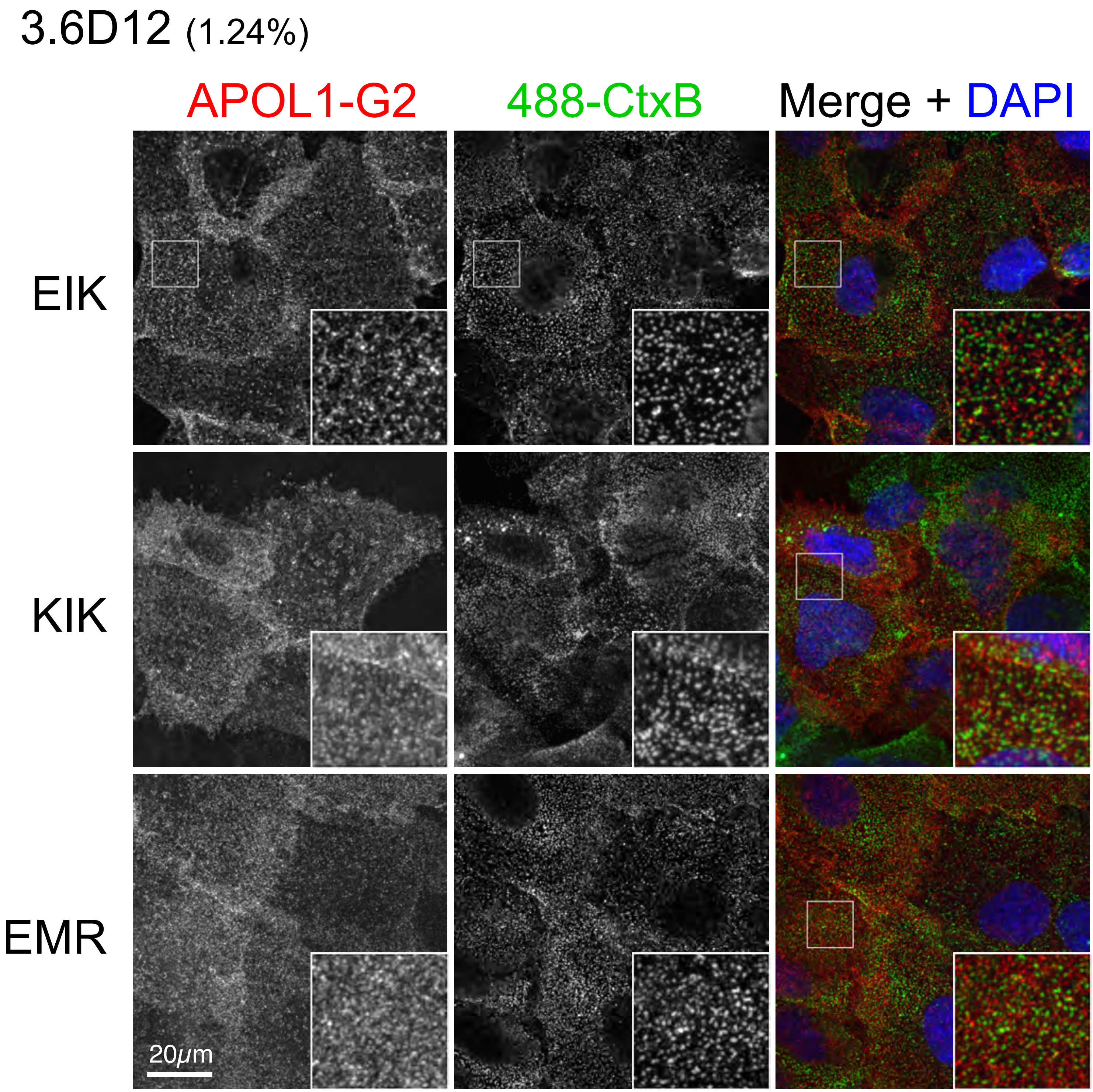

Figure S21. APOL1 clusters are not in lipid rafts stained for GM1.
Different images of 3.6D12 (1.24% aggregated) anti-APOL1-incubated iAPOL1-G2-EIK podocytes from the same experiment as in Figure 7 are shown on the left (red channel), with cross-linked (for 15' at 37°C) Alexa488-Cholera Toxin B (green), which binds the ganglioside GM1, which partitions into lipid rafts and caveolae (center). Overlays with nuclear DAPI (blue) are on the right. Insets are 3x magnifications of the boxes. Neither the large (EIK) nor small (KIK and EMR) clusters induced by 3.6D12 overlap with GM1. Antibody 4.2C4-mediated clusters similarly anti-correlate with lipid rafts (data not shown).

a

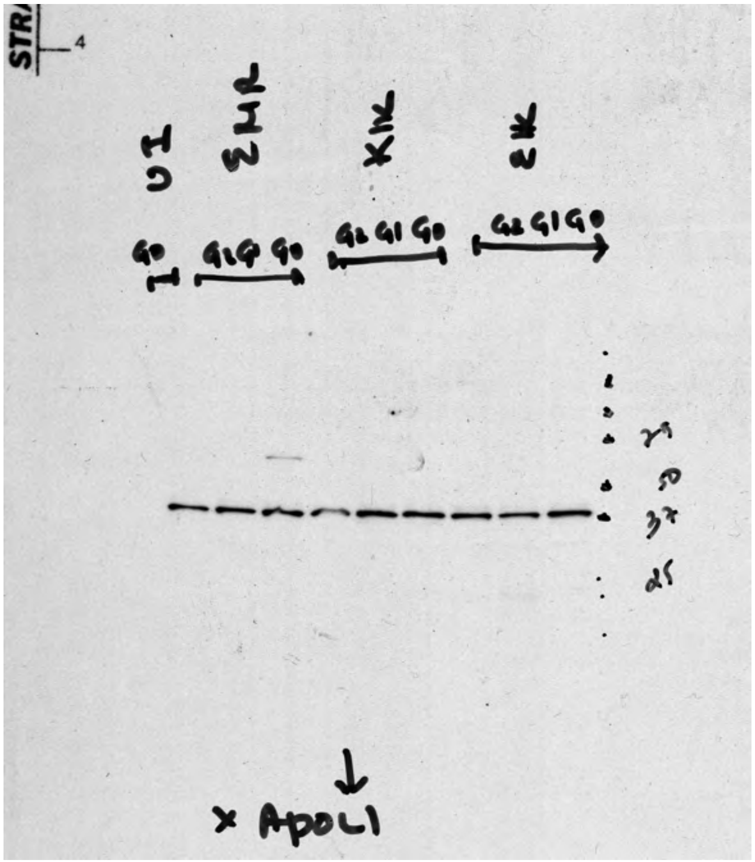

b

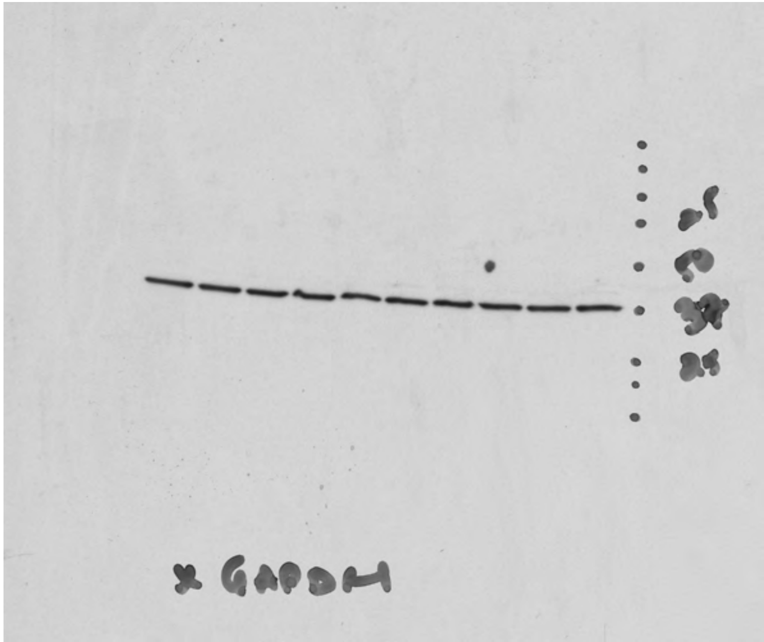

Figure S22. Full length APOL1/GAPDH Western blots for Figure 2b (of all iAPOL1.vA haplotypes).
(a) APOL1 western blot of the 9 iAPOL1 podocyte cell lines used in Fig. 2b (scanned back to front, so in opposite order to the figure). The antibody was a mixture of 3.1C1 and 3.7D6 (ref. 31) and did not produce any other bands than those shown.
(b) GAPDH loading control for Fig. 2b (also scanned back to front). Rabbit monoclonal EPR16891 (0.93µg/ml Ab181602, lot GR217575-61) was used for this blot and exhibited no bands other than those shown. The empty parts of the films outside of the blots were not saved in the scanned file to save file space.

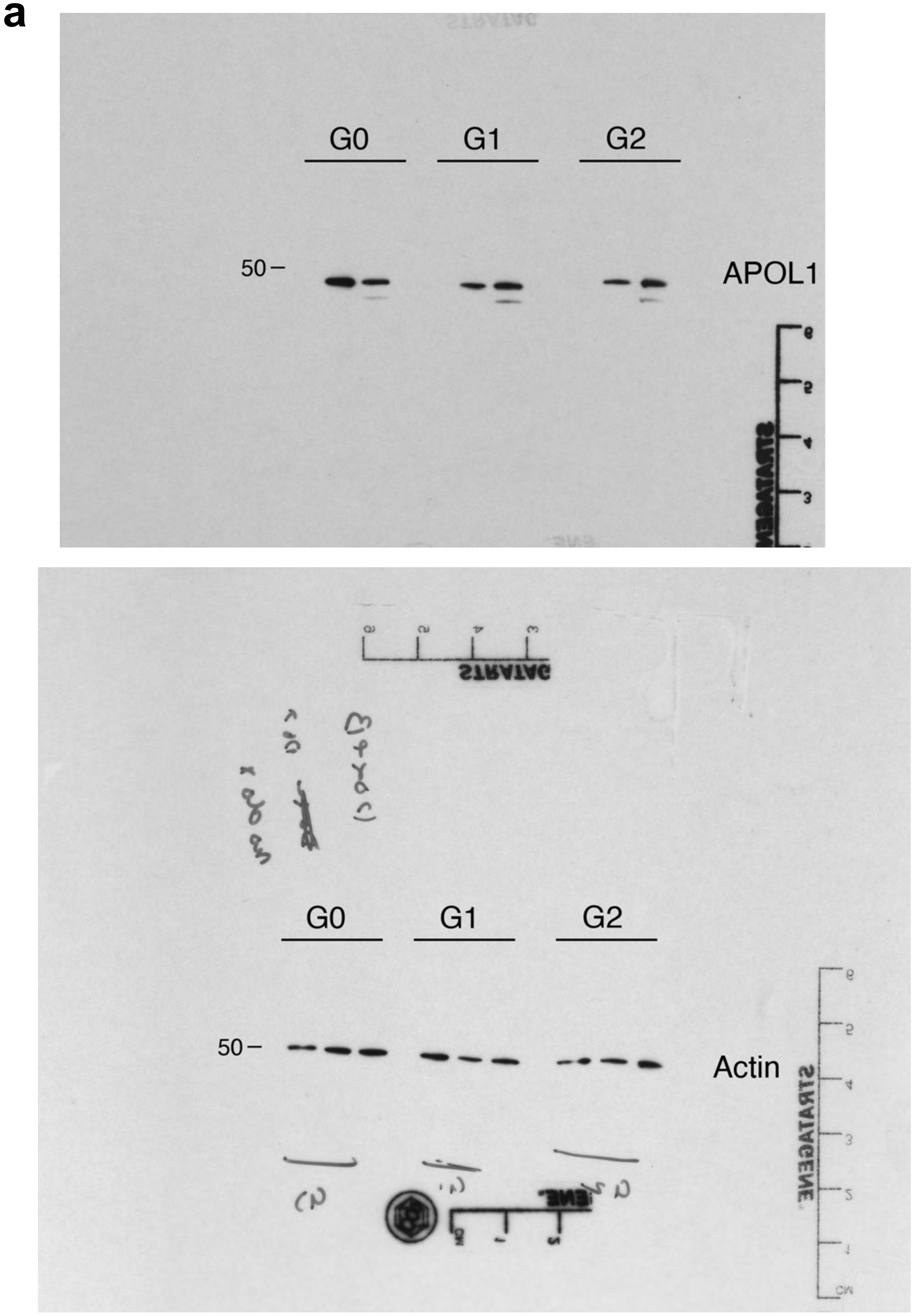

Figure S23. Full length APOL1/actin Western blots for Figure 5b (BFA-treated iAPOL1.vA podocytes)
(a) Full APOL1 western blot of the BFA-treated vA podocytes in Fig. 5b (same orientation as in the figure). The same rabbit monoclonals 3.1C1 and 3.7D6 (0.05µg/ml) were used (ref. 19).
(b) Actin loading controls for Fig. 5b. Note an empty lane was run in between the variants on the same blot. Empty parts of the films with no signals were cropped before saving the scan.

a

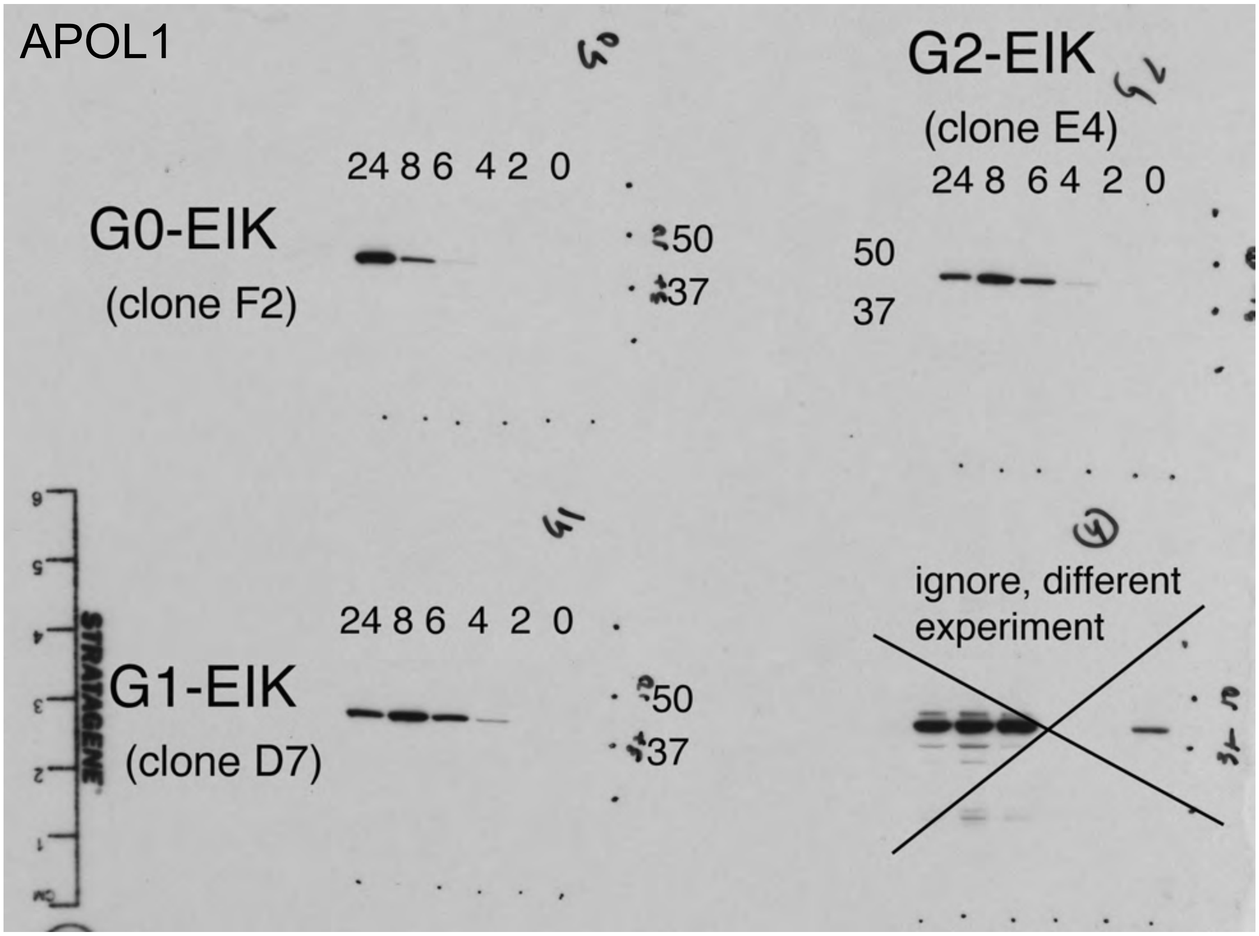

b

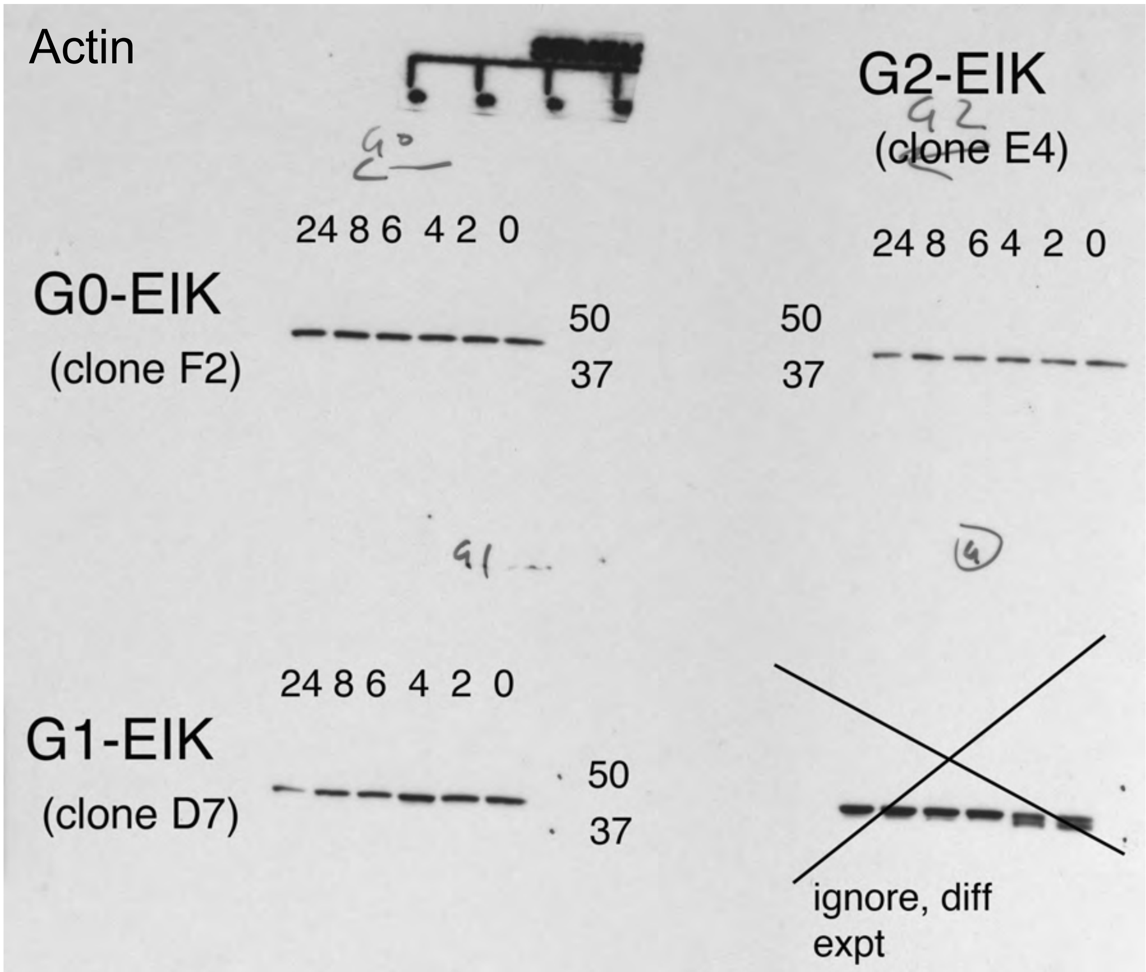

Figure S24. Full length APOL1/actin Western blots for Supplementary Figure 9a (dox time course). (a) Full APOL1 western blot of the 5ng/ml Dox time-course shown in Fig. S9a (back to front compared to the figure). (b) Actin loading controls for Fig. S9a (also scanned back to front). Both panels were shrunk 90% to fit the page. The blank parts of the films outside the blots were cropped to save file space, so the film edges are not visible.

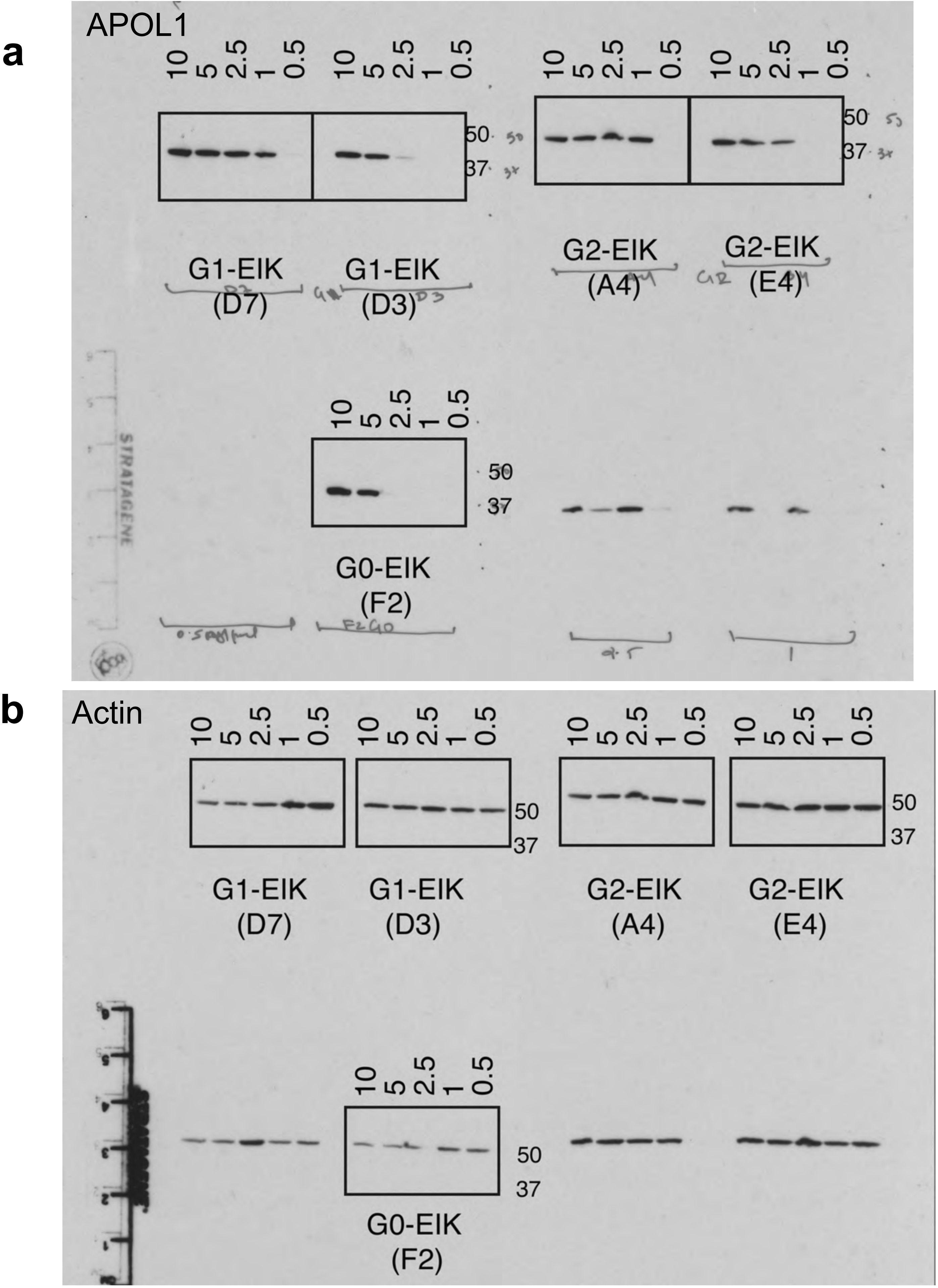

Figure S25. Full length APOL1/actin Western blots for Supplementary Figure 9b.
(a) Full APOL1 western blot of the Dox titration shown in Fig. S9b (back to front compared to the figure).The blots without boxes around them were not used in the figure. Two sets of clones were run on each blot.
(b) Actin loading controls for Fig. S9b (also scanned back to front). Both panels shrunk 90% to fit page. The blank parts of the films outside of the blots were cropped to save file space, so the film edges are not visible.

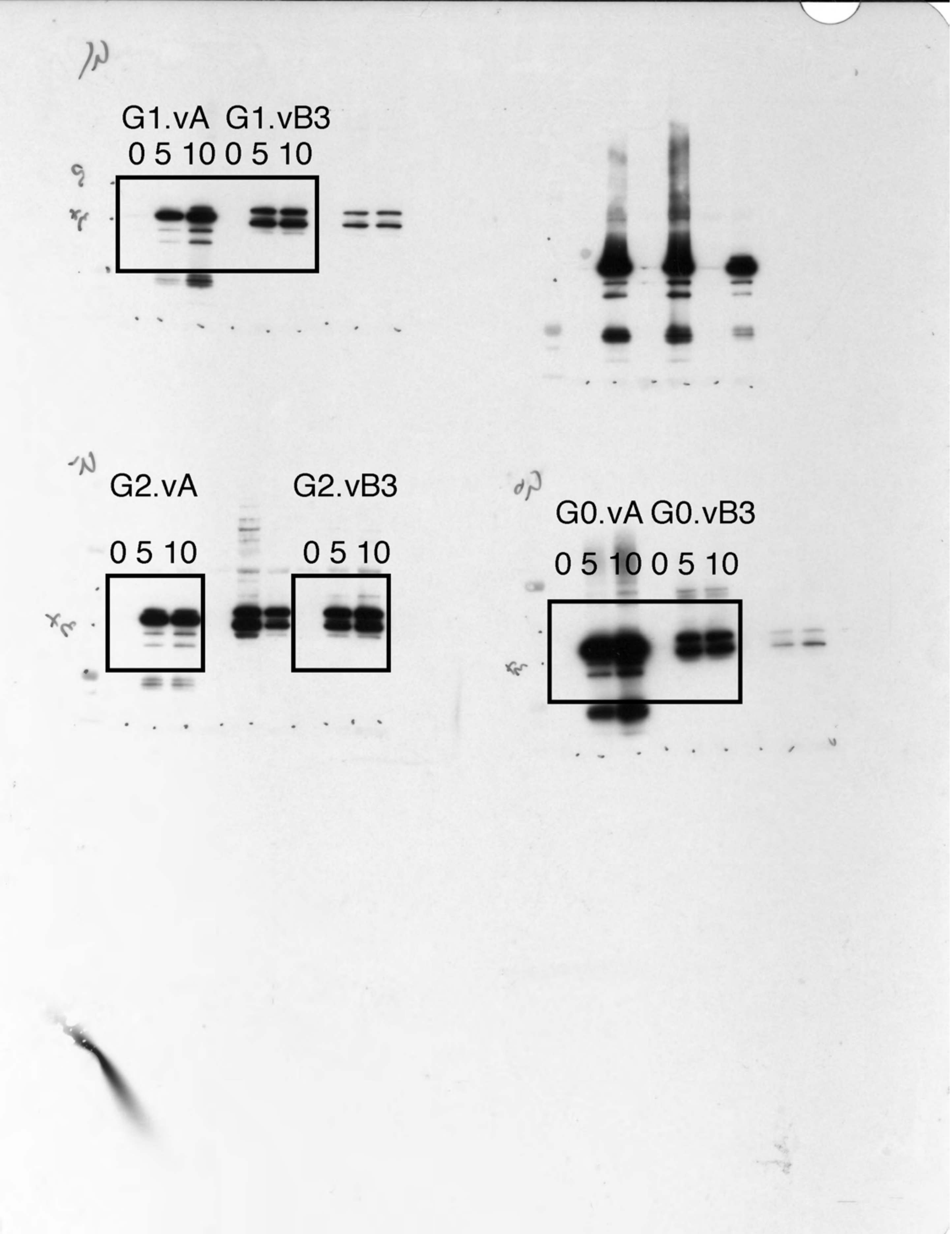

Figure S26. Full length APOL1 Western Blots for Supplementary Figure 13a (iAPOL1.vB3 podocytes). Black boxes indicate the regions cut and pasted into Supplementary Fig. 13a. The unused bands indicate other clones that did not express as highly as the chosen vB3 clones. Lead iAPOL1.vA isoforms were run on each blot for direct comparison of expression levels. The top of the film is visible, but the empty bottom and sides were cropped.

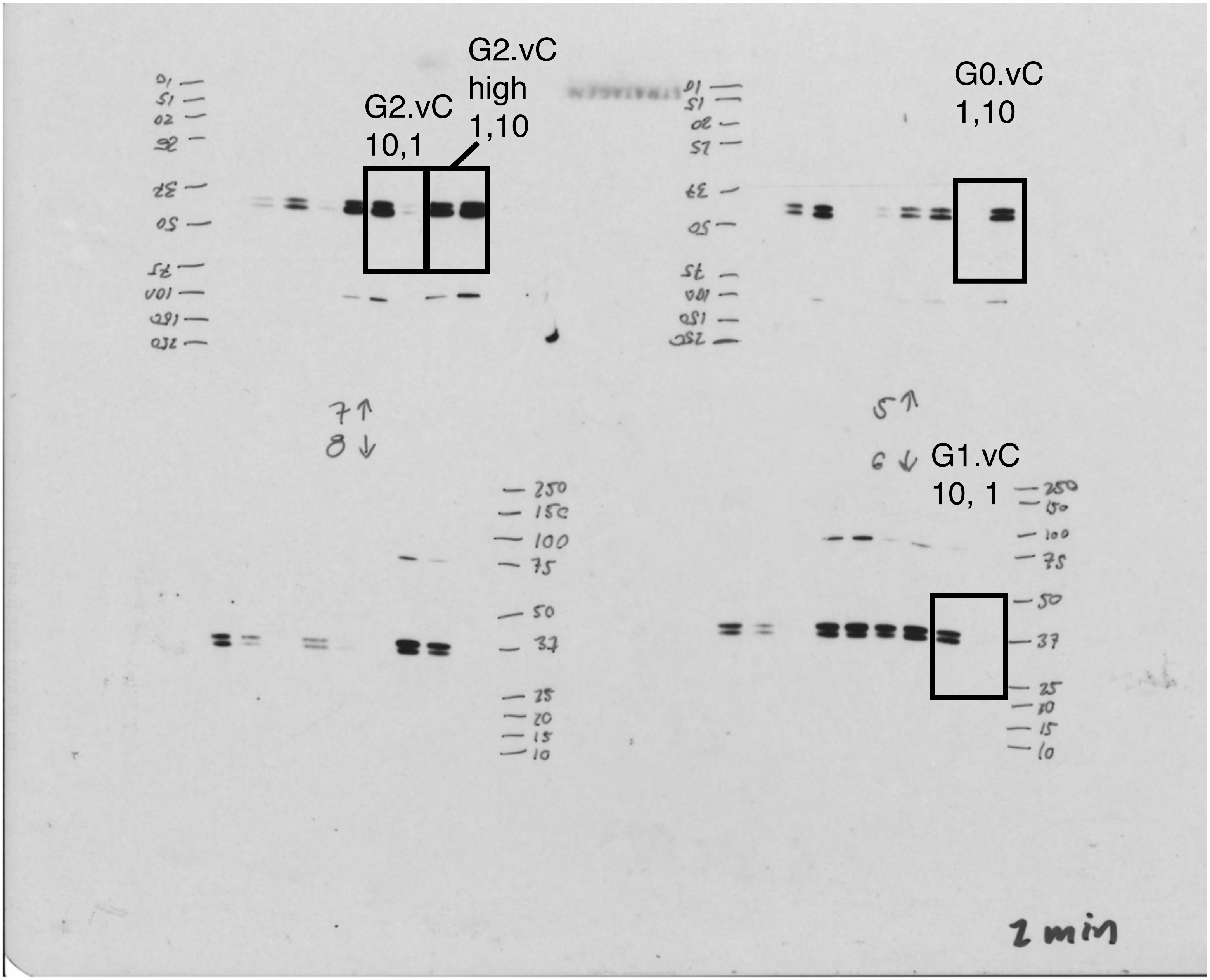

Figure S27. Full length APOL1 Western blots for Supplementary Figure 13b (iAPOL1.vC podocytes).
The black boxes indicate the clones cut and pasted into Fig. S13b. Note that G0.vC and G2.vC blots were scanned upside down and that, where necessary, lanes were flipped so that 1ng/ml precedes 10ng/ml dox in the final figure. The edges of the film are visible on the left and bottom, but were cropped off on the top and right without deleting any information.
