# Supplementary material for: Apolipoprotein L1 (APOL1) renal risk variant-mediated podocyte cytotoxicity depends on African haplotype and surface expression
Source: Sci Rep. 2024 Feb 14;14:3765. doi: 10.1038/s41598-024-53298-4 (PMC10866943; doi:10.1038/s41598-024-53298-4)
Supplement: Supplementary file 2 — Supplementary Table S1. [file 41598_2024_53298_MOESM2_ESM.docx]

| Name | Amino acid sequence |
| --- | --- |
| G0-EMR^1^ | MEGAALLRVSVLCIWMSALFLGVGVRAEEAGARVQQNVPSGTDTGDPQSKPLGDWAAGTMDPESSIFIEDAIKYFKEKVSTQNLLLLLTDNEAWNGFVAAAELPRNEADELRKALDNLARQMIMKDKNWHDKGQQYRNWFLKEFPRLKSELEDNIRRLRALADGVQKVHKGTTIANVVSGSLSISSGILTLVGMGLAPFTEGGSLVLLEPGMELGITAALTGITSSTMDYGKKWWTQAQAHDLVIKSLDKLKEVREFLGENISNFLSLAGNTYQLTRGIGKDIRALRRARANLQSVPHASASRPRVTEPISAESGEQVERVNEPSILEMSRGVKLTDVAPVSFFLVLDVVYLVYESKHLHEGAKSETAEELKKVAQELEEKLNILNNNYKILQADQEL |
| G1-EMR | MEGAALLRVSVLCIWMSALFLGVGVRAEEAGARVQQNVPSGTDTGDPQSKPLGDWAAGTMDPESSIFIEDAIKYFKEKVSTQNLLLLLTDNEAWNGFVAAAELPRNEADELRKALDNLARQMIMKDKNWHDKGQQYRNWFLKEFPRLKSELEDNIRRLRALADGVQKVHKGTTIANVVSGSLSISSGILTLVGMGLAPFTEGGSLVLLEPGMELGITAALTGITSSTMDYGKKWWTQAQAHDLVIKSLDKLKEVREFLGENISNFLSLAGNTYQLTRGIGKDIRALRRARANLQSVPHASASRPRVTEPISAESGEQVERVNEPSILEMSRGVKLTDVAPVGFFLVLDVVYLVYESKHLHEGAKSETAEELKKVAQELEEKLNMLNNNYKILQADQEL |
| G2-EMR | MEGAALLRVSVLCIWMSALFLGVGVRAEEAGARVQQNVPSGTDTGDPQSKPLGDWAAGTMDPESSIFIEDAIKYFKEKVSTQNLLLLLTDNEAWNGFVAAAELPRNEADELRKALDNLARQMIMKDKNWHDKGQQYRNWFLKEFPRLKSELEDNIRRLRALADGVQKVHKGTTIANVVSGSLSISSGILTLVGMGLAPFTEGGSLVLLEPGMELGITAALTGITSSTMDYGKKWWTQAQAHDLVIKSLDKLKEVREFLGENISNFLSLAGNTYQLTRGIGKDIRALRRARANLQSVPHASASRPRVTEPISAESGEQVERVNEPSILEMSRGVKLTDVAPVSFFLVLDVVYLVYESKHLHEGAKSETAEELKKVAQELEEKLNILNN--KILQADQEL |
| G0-EIK | MEGAALLRVSVLCIWMSALFLGVGVRAEEAGARVQQNVPSGTDTGDPQSKPLGDWAAGTMDPESSIFIEDAIKYFKEKVSTQNLLLLLTDNEAWNGFVAAAELPRNEADELRKALDNLARQMIMKDKNWHDKGQQYRNWFLKEFPRLKSELEDNIRRLRALADGVQKVHKGTTIANVVSGSLSISSGILTLVGMGLAPFTEGGSLVLLEPGMELGITAALTGITSSTIDYGKKWWTQAQAHDLVIKSLDKLKEVKEFLGENISNFLSLAGNTYQLTRGIGKDIRALRRARANLQSVPHASASRPRVTEPISAESGEQVERVNEPSILEMSRGVKLTDVAPVSFFLVLDVVYLVYESKHLHEGAKSETAEELKKVAQELEEKLNILNNNYKILQADQEL |
| G1-EIK | MEGAALLRVSVLCIWMSALFLGVGVRAEEAGARVQQNVPSGTDTGDPQSKPLGDWAAGTMDPESSIFIEDAIKYFKEKVSTQNLLLLLTDNEAWNGFVAAAELPRNEADELRKALDNLARQMIMKDKNWHDKGQQYRNWFLKEFPRLKSELEDNIRRLRALADGVQKVHKGTTIANVVSGSLSISSGILTLVGMGLAPFTEGGSLVLLEPGMELGITAALTGITSSTIDYGKKWWTQAQAHDLVIKSLDKLKEVKEFLGENISNFLSLAGNTYQLTRGIGKDIRALRRARANLQSVPHASASRPRVTEPISAESGEQVERVNEPSILEMSRGVKLTDVAPVGFFLVLDVVYLVYESKHLHEGAKSETAEELKKVAQELEEKLNMLNNNYKILQADQEL |
| G2-EIK | MEGAALLRVSVLCIWMSALFLGVGVRAEEAGARVQQNVPSGTDTGDPQSKPLGDWAAGTMDPESSIFIEDAIKYFKEKVSTQNLLLLLTDNEAWNGFVAAAELPRNEADELRKALDNLARQMIMKDKNWHDKGQQYRNWFLKEFPRLKSELEDNIRRLRALADGVQKVHKGTTIANVVSGSLSISSGILTLVGMGLAPFTEGGSLVLLEPGMELGITAALTGITSSTIDYGKKWWTQAQAHDLVIKSLDKLKEVKEFLGENISNFLSLAGNTYQLTRGIGKDIRALRRARANLQSVPHASASRPRVTEPISAESGEQVERVNEPSILEMSRGVKLTDVAPVSFFLVLDVVYLVYESKHLHEGAKSETAEELKKVAQELEEKLNILNN--KILQADQEL |

| G0-KIK | MEGAALLRVSVLCIWMSALFLGVGVRAEEAGARVQQNVPSGTDTGDPQSKPLGDWAAGTMDPESSIFIEDAIKYFKEKVSTQNLLLLLTDNEAWNGFVAAAELPRNEADELRKALDNLARQMIMKDKNWHDKGQQYRNWFLKEFPRLKSKLEDNIRRLRALADGVQKVHKGTTIANVVSGSLSISSGILTLVGMGLAPFTEGGSLVLLEPGMELGITAALTGITSSTIDYGKKWWTQAQAHDLVIKSLDKLKEVKEFLGENISNFLSLAGNTYQLTRGIGKDIRALRRARANLQSVPHASASRPRVTEPISAESGEQVERVNEPSILEMSRGVKLTDVAPVSFFLVLDVVYLVYESKHLHEGAKSETAEELKKVAQELEEKLNILNNNYKILQADQEL |
| --- | --- |
| G1-KIK | MEGAALLRVSVLCIWMSALFLGVGVRAEEAGARVQQNVPSGTDTGDPQSKPLGDWAAGTMDPESSIFIEDAIKYFKEKVSTQNLLLLLTDNEAWNGFVAAAELPRNEADELRKALDNLARQMIMKDKNWHDKGQQYRNWFLKEFPRLKSKLEDNIRRLRALADGVQKVHKGTTIANVVSGSLSISSGILTLVGMGLAPFTEGGSLVLLEPGMELGITAALTGITSSTIDYGKKWWTQAQAHDLVIKSLDKLKEVKEFLGENISNFLSLAGNTYQLTRGIGKDIRALRRARANLQSVPHASASRPRVTEPISAESGEQVERVNEPSILEMSRGVKLTDVAPVGFFLVLDVVYLVYESKHLHEGAKSETAEELKKVAQELEEKLNMLNNNYKILQADQEL |
| G2-KIK | MEGAALLRVSVLCIWMSALFLGVGVRAEEAGARVQQNVPSGTDTGDPQSKPLGDWAAGTMDPESSIFIEDAIKYFKEKVSTQNLLLLLTDNEAWNGFVAAAELPRNEADELRKALDNLARQMIMKDKNWHDKGQQYRNWFLKEFPRLKSKLEDNIRRLRALADGVQKVHKGTTIANVVSGSLSISSGILTLVGMGLAPFTEGGSLVLLEPGMELGITAALTGITSSTIDYGKKWWTQAQAHDLVIKSLDKLKEVKEFLGENISNFLSLAGNTYQLTRGIGKDIRALRRARANLQSVPHASASRPRVTEPISAESGEQVERVNEPSILEMSRGVKLTDVAPVSFFLVLDVVYLVYESKHLHEGAKSETAEELKKVAQELEEKLNILNN--KILQADQEL |
| G0.vC- EIK | MEGAALLRVSVLCIW------------------VQQNVPSGTDTGDPQSKPLGDWAAGTMDPESSIFIEDAIKYFKEKVSTQNLLLLLTDNEAWNGFVAAAELPRNEADELRKALDNLARQMIMKDKNWHDKGQQYRNWFLKEFPRLKSELEDNIRRLRALADGVQKVHKGTTIANVVSGSLSISSGILTLVGMGLAPFTEGGSLVLLEPGMELGITAALTGITSSTIDYGKKWWTQAQAHDLVIKSLDKLKEVKEFLGENISNFLSLAGNTYQLTRGIGKDIRALRRARANLQSVPHASASRPRVTEPISAESGEQVERVNEPSILEMSRGVKLTDVAPVSFFLVLDVVYLVYESKHLHEGAKSETAEELKKVAQELEEKLNILNNNYKILQADQEL |
| G1.vC-EIK | MEGAALLRVSVLCIW------------------VQQNVPSGTDTGDPQSKPLGDWAAGTMDPESSIFIEDAIKYFKEKVSTQNLLLLLTDNEAWNGFVAAAELPRNEADELRKALDNLARQMIMKDKNWHDKGQQYRNWFLKEFPRLKSELEDNIRRLRALADGVQKVHKGTTIANVVSGSLSISSGILTLVGMGLAPFTEGGSLVLLEPGMELGITAALTGITSSTIDYGKKWWTQAQAHDLVIKSLDKLKEVKEFLGENISNFLSLAGNTYQLTRGIGKDIRALRRARANLQSVPHASASRPRVTEPISAESGEQVERVNEPSILEMSRGVKLTDVAPVGFFLVLDVVYLVYESKHLHEGAKSETAEELKKVAQELEEKLNMLNNNYKILQADQEL |
| G2.vC-EIK | MEGAALLRVSVLCIW------------------VQQNVPSGTDTGDPQSKPLGDWAAGTMDPESSIFIEDAIKYFKEKVSTQNLLLLLTDNEAWNGFVAAAELPRNEADELRKALDNLARQMIMKDKNWHDKGQQYRNWFLKEFPRLKSELEDNIRRLRALADGVQKVHKGTTIANVVSGSLSISSGILTLVGMGLAPFTEGGSLVLLEPGMELGITAALTGITSSTIDYGKKWWTQAQAHDLVIKSLDKLKEVKEFLGENISNFLSLAGNTYQLTRGIGKDIRALRRARANLQSVPHASASRPRVTEPISAESGEQVERVNEPSILEMSRGVKLTDVAPVSFFLVLDVVYLVYESKHLHEGAKSETAEELKKVAQELEEKLNILNN--KILQADQEL |

| G0.vB3-EIK | MRFKSHTVELRRPCSDMEGAALLRVSVLCIW------------------VQQNVPSGTDTGDPQSKPLGDWAAGTMDPESSIFIEDAIKYFKEKVSTQNLLLLLTDNEAWNGFVAAAELPRNEADELRKALDNLARQMIMKDKNWHDKGQQYRNWFLKEFPRLKSELEDNIRRLRALADGVQKVHKGTTIANVVSGSLSISSGILTLVGMGLAPFTEGGSLVLLEPGMELGITAALTGITSSTIDYGKKWWTQAQAHDLVIKSLDKLKEVKEFLGENISNFLSLAGNTYQLTRGIGKDIRALRRARANLQSVPHASASRPRVTEPISAESGEQVERVNEPSILEMSRGVKLTDVAPVSFFLVLDVVYLVYESKHLHEGAKSETAEELKKVAQELEEKLNILNNNYKILQADQEL |
| --- | --- |
| G1.vB3-EIK | MRFKSHTVELRRPCSDMEGAALLRVSVLCIW------------------VQQNVPSGTDTGDPQSKPLGDWAAGTMDPESSIFIEDAIKYFKEKVSTQNLLLLLTDNEAWNGFVAAAELPRNEADELRKALDNLARQMIMKDKNWHDKGQQYRNWFLKEFPRLKSELEDNIRRLRALADGVQKVHKGTTIANVVSGSLSISSGILTLVGMGLAPFTEGGSLVLLEPGMELGITAALTGITSSTIDYGKKWWTQAQAHDLVIKSLDKLKEVKEFLGENISNFLSLAGNTYQLTRGIGKDIRALRRARANLQSVPHASASRPRVTEPISAESGEQVERVNEPSILEMSRGVKLTDVAPVGFFLVLDVVYLVYESKHLHEGAKSETAEELKKVAQELEEKLNMLNNNYKILQADQEL |
| G2.vB3-EIK | MRFKSHTVELRRPCSDMEGAALLRVSVLCIW------------------VQQNVPSGTDTGDPQSKPLGDWAAGTMDPESSIFIEDAIKYFKEKVSTQNLLLLLTDNEAWNGFVAAAELPRNEADELRKALDNLARQMIMKDKNWHDKGQQYRNWFLKEFPRLKSELEDNIRRLRALADGVQKVHKGTTIANVVSGSLSISSGILTLVGMGLAPFTEGGSLVLLEPGMELGITAALTGITSSTIDYGKKWWTQAQAHDLVIKSLDKLKEVKEFLGENISNFLSLAGNTYQLTRGIGKDIRALRRARANLQSVPHASASRPRVTEPISAESGEQVERVNEPSILEMSRGVKLTDVAPVSFFLVLDVVYLVYESKHLHEGAKSETAEELKKVAQELEEKLNILNN--KILQADQEL |

^1^APOL1-G0 EMR is RefSeq NM_003661. Unless otherwise stated the sequences refer to isoform vA.

E150K (rs2239785), M228I (rs136175) and R255K (rs136176) are in magenta; G1 (S342G/I384M; rs73885319/rs60910145)

are in red; the 2aa deleted in G2 (N388, Y389; rs71785313) are in blue or blue dashes where deleted.

The 18aa encoded by exon 4 in vA are in orange (or dashes to denote their absence from isoforms vB3 and vC) and the

extra 16aa encoded by exon 2 in vB3 are highlighted in yellow. The 27aa signal sequence in vA is underlined.
